# Investigating the two regimes of fibrin clot lysis: an experimental and computational approach

**DOI:** 10.1016/j.bpj.2021.08.005

**Published:** 2021-08-10

**Authors:** Franck Raynaud, Alexandre Rousseau, Daniel Monteyne, David Perez-Morga, Karim Zouaoui Boudjeltia, Bastien Chopard

**Affiliations:** 1 Department of Computer Science, University of Geneva, Geneva, Switzerland; 2 Laboratoire de Médecine Expérimentale, Medicine Faculty, Université libre de Bruxelles (ULB 222 Unit), ISPPC CHU de Charleroi, Hôpital A. Vésale, Montigny-le-Tilleul, Belgium; 3 Laboratory of Molecular Parasitology, IBMM, Université libre de Bruxelles, Gosselies, Belgium; 4 Center for Microscopy and Molecular Imaging, Université libre de Bruxelles, Gosselies, Belgium

## Abstract

It has been observed in vitro that complete clot lysis is generally preceded by a slow phase of lysis during which the degradation seems to be inefficient. However, this slow regime was merely noticed, but not yet quantitatively discussed. In our experiments, we observed that the lysis ubiquitously occurred in two distinct regimes, a slow and a fast lysis regime. We quantified extensively the duration of these regimes for a wide spectrum of experimental conditions and found that on average, the slow regime lasts longer than the fast one, meaning that during most of the process, the lysis is ineffective. We proposed a computational model in which the properties of the binding of the proteins change during the lysis: first, the biochemical reactions take place at the surface of the fibrin fibers, then in the bulk, resulting in the observed fast lysis regime. This simple hypothesis appeared to be sufficient to reproduce with a great accuracy the lysis profiles obtained experimentally.

## Significance

Although the interplay between the main components of the fibrinolytic system is well understood, some dynamical aspects of the fibrinolysis remain unclear. Notably, we observe that in vitro, fibrin-rich clots undergo a slow and inefficient phase of degradation when subject to endogenous fibrinolysis. In fact, it turns out that a large part of the lysis process operates in this slow regime. To explain this observation, we proposed a computational model in which the properties of the binding of the proteins change during the lysis. First, plasminogen and tissue plasminogen activator bind at the surface of the fibers, resulting in a slow lysis, then in the bulk of the fibers, thus speeding up the degradation of the clot.

## Introduction

Fibrinolysis is the process by which a thrombus is dissolved. The fibrinolytic system removes the fibrin clot once it has achieved its hemostatic function. By nature, fibrinolysis is delayed, at least relative to coagulation: fibrin is formed in a matter of minutes or even seconds, generally serving a useful purpose, such as the healing of a breach in the vascular wall; afterwards, it has to be removed over a period of hours or days, as in the meantime, tissue repair can take place. Fibrinolysis uses elements from plasma, platelets, tissue, and other blood cells to regulate the degradation of fibrin. This is induced by the conversion of plasminogen (Pg), a plasma protein that circulates as a zymogen to the serine protease, plasmin. Plasmin is a trypsin-like enzyme that cleaves a wide variety of plasma proteins. However, many of these reactions require extremely high levels of plasmin. The physiological function of plasmin is limited primarily to the degradation of the fibrin clot and extracellular matrix molecules. The conversion of Pg to plasmin is achieved by a variety of plasminogen activators that exist in tissues throughout the body (1). The plasminogen activators that are important in this process include tissue-type plasminogen activator and urokinase-type plasminogen activator. Several studies have reported that the risk of ischemic cardiovascular events is increased in patients with impaired fibrinolytic function (2, 3, 4, 5). Fibrinolytic activity is primarily determined by the balance between the levels of tissue plasminogen activator (tPA), plasminogen activator inhibitor 1 (PAI-1), and the plasmin inhibitor *α*2-antiplasmin. The endothelial cells are responsible for the production and blood release of tPA and of PAI-1. Multiple factors such as lipoproteins, cytokines, and inflammatory markers modulate endothelial cells to produce tPA and PAI-1 (6). In 2016, the World Health Organization identified stroke as the second cause of death for both sexes and across all ages (7). Clinical interventions can be classified into either chemical (thrombolysis) or mechanical (thrombectomy) treatment. In particular, a combination of both appeared to be a promising approach regarding patient outcomes (8,9). However, the time window for the treatment at the onset of the stroke remains the most critical parameter, and patients who benefited from early intervention are associated with significantly better 3-month prognosis (10), but treatment success is highly variable (11). Meanwhile, a large effort is encouraging in silico trials for thrombectomy and thrombolysis (12). A better understanding of the origin of the variability of treatment success is still an issue from the clinical perspective and also remains a critical challenge from a modeling point of view. The blood coagulation cascade and fibrinolysis process are striking examples of processes that are more complex than the sum of their parts. Each component (including fibrin polymerization and fibrinolysis) is well understood when considered individually, isolated from other parts. However, taken as a whole, hemodynamics, nonlinearities, feedback loops, and the variability in physiological conditions dramatically increase the complexity of the coagulation system. Even though it is recognized that computer simulations are an essential tool to deal with this complexity, mathematical modeling remains challenging. Compartmental models describing the biochemical processes of the coagulation and the lysis of the clot require dozens of differential equations (13,14), as well as advanced sensitivity analysis, to decipher the most relevant components of the reactions (15,16).

In this work, we combine in vitro experiments and computational modeling analysis of fibrin-rich clot formation and lysis. The dynamics of the processes were monitored from the time of clot formation to the time of complete lysis. A computerized semiautomatic device based on turbidity measurements was used to determine the amount of fibrin present in the system. We investigated the effect of changing the concentration of the main components of the fibrinolytic system (fibrinogen, Pg, tPA, and PAI-1) on the clot formation and lysis times. Analysis of turbidity time curves reveals that for all experimental conditions, the lysis of fibrin-rich clots is characterized by two regimes: a slow regime in which the change in turbidity is linear, followed by a regime with faster turbidity change. The presence of two regimes of lysis was already visible in several experimental studies such as plasma clot lysis with addition of vessel wall components (17), euglobulin lysis from different types of donors (18), fibrin clots formed with positively charged peptides (19), and plasma clot lysis of normal and cirrhotic patients (20). Phase of latency was also indicated as part of the lysis in (2,21). These works investigated lysis times for various experimental conditions; however, the distinction between a slow and a fast regime of lysis was not discussed. The goal of our computational model is to propose a mechanism explaining the slow regime observed in our experiments. We proposed the hypothesis that the slow (respectively (resp.) fast) regime corresponded to lysis kinetics occurring at the surface (resp. in the bulk) of the fibers. Our results indicated that to best reproduce the experimental lysis curves, the “depth” of the reactions has to increase as the lysis proceeds from the surface to eventually the entire bulk of the fibers, that is to say for all the protofibrils in the fiber. We compared our hypothesis against a model with heterogeneous cross-sectional distribution of protofibrils (22), and finally, we proposed a surrogate model for the transition between the slow and fast regimes that appeared to be more accurate in predicting lysis times compared to models that consider either surface or bulk reactions.

## Materials and methods

### Experimental procedures and data acquisition

**Figure 1:**
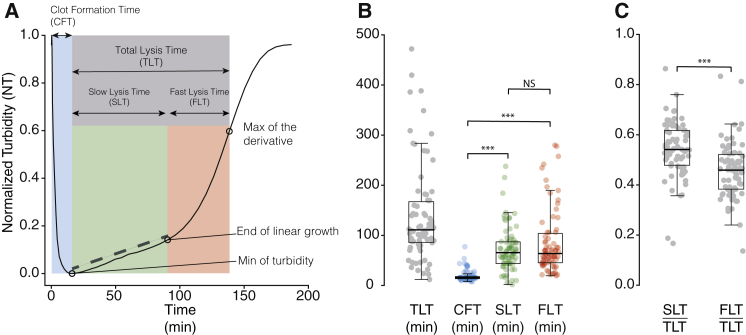
Typical experiment of fibrinolysis and timescales of the processes. (*A*) Normalized turbidity curve of a fibrinolysis experiment showing the time course of the normalized turbidity (NT) with the formation of the clot (*blue*), the total fibrinolysis (*gray*), and the slow (*green*) and fast (*red*) regimes. (*B*) Total lysis times (TLTs), clot formation times (CFTs), slow lysis times (SLTs), and fast lysis times (FLTs) for all experiments. (*C*) Ratio of SLTs and FLTs/TLT. ^∗∗∗^*p* < 10^−4^. The boxplots represent the median as well as the first and third quartiles (*B* and *C*). To see this figure in color, go online.

Our experiment consisted of mixing fibrinogen, Pg, tPA, and PAI-1 in various concentrations in a test tube. Then, an excess of thrombin was added, and the attenuation of a light beam through the test tube was measured as a function of time, first indicating the clot formation and then the clot lysis. For this purpose, we designed a completely computerized semiautomated eight-channel measurement and determination of fibrin clot lysis (EREM, Charleroi, Belgium) (21). Briefly, a computer records the data from each channel every 1 min. A software generates the graph of the clot formation and fibrinolytic process. The design of a lysis curve is exemplified in Fig. 1 *A*. The *x* axis and *y* axis, respectively, represent time and evolution of the signal sensor. A block is made of pure aluminum (360 × 30 × 100 mm) and is warmed by two resistances of 20 W. It is designed to insert spectrophotometric microcuvettes (10 × 4 × 45 mm; Sarstedt, Nümbrecht, Germany) into eight wells. Each well includes one emitter (diode: SFH 409) and one receptor (phototransistor: SFH 309FA), both operating at 890 nm. When the clot is formed, the phototransistor signal decreases, reflecting a Tyndall effect. For clot formation and lysis analysis, we used the purified proteins tPA (human recombinant; Hyphen Biomed, Paris, France), PAI-1 (human recombinant; Sigma-Aldrich, Darmstadt, Germany), Pg (human plasma purified protein; Sigma-Aldrich), and fibrinogen (human plasma purified protein; Sigma-Aldrich). Experimental conditions are presented in Table 1.

**Table 1:** Summary table of the different experimental conditions

Label	Fibrinogen (mg ⋅ mL^−1^)	tPA (*μ*g ⋅ mL^−1^)	PAI-1 (*μ*g ⋅ mL^−1^)	Pg (*μ*g ⋅ mL^−1^)	Number of individual experiments
1	3	0.0005	0.01	13	4
2	3	0.001	0.01	13	18 (control case)
3	3	0.002	0.01	13	4
4	3	0.004	0.01	13	4
5	3	0.008	0.01	13	4
6	3	0.001	0.0025	13	4
7	3	0.001	0.005	13	4
8	3	0.001	0.02	13	4
9	3	0.001	0.04	13	2
10	3	0.001	0.01	2.6	3
11	3	0.001	0.01	5.2	4
12	3	0.001	0.01	26	4
13	3	0.001	0.01	52	4
14	0.8	0.001	0.01	13	2
15	1	0.001	0.01	13	2
16	2	0.001	0.01	13	2
17	4	0.001	0.01	13	2
18	5	0.001	0.01	13	1
19	6	0.001	0.01	13	2
					total = 74

Experiments 1 and 3–13 were made with four replicates and experiments 14–19 with two replicates (different values indicate that some experiments could not be used for the analysis). Experiment 2 was our control case and had more replicates.

We used values for tPA between 0.0005 and 0.008 *μ*g ⋅ mL^−1^. This range covered the physiological value of 0.005 *μ*g ⋅ mL^−1^. In our experiments, the total lysis time decreased rapidly with the concentration of tPA. To not overwhelm the effect of the other molecules, we used a default value of tPA, 0.001 *μ*g ⋅ mL^−1^, lower than the physiological concentration. Pg values were lower than physiological conditions. However, in vivo Pg must be high enough to compensate the presence of *α*2-antiplasmin. In our experiments, we observed that the effect saturated for concentrations of Pg greater than 13 *μ*g ⋅ mL^−1^, suggesting that we covered a sufficiently large range of values for Pg. The default concentrations of PAI-1 and fibrinogen have been chosen near their physiological concentrations (23,24). These values were at the center of the linear dose-response relationship between total lysis time and concentrations of PAI-1 and fibrinogen, hence allowing us to explore the behavior of our experimental system for various concentrations around the default values.

### Normalization of experimental data

To compare lysis curves from different experimental conditions, we transformed the original turbidity curve into normalized turbidity (NT) as follows:

1) Original data were sliced, and we retained one time step over three to discard the fluctuations of the signal between two consecutive time points.
2) Sliced data were then smoothed using the Savitzky-Golay filter (from the signal processing scipy.signal library) with a window of five points before and after the point of interest. Following the standard good practice with the Savitzky-Golay filter for signals with change in concavity, we used a third-order polynomial fit.
3) Smoothed data were then normalized by the maximal value of the turbidity after subtracting the minimal value.

This procedure resulted in values of NT between 0 and 1 and allowed us to compare different experimental conditions. This step of normalization was necessary as we found that the maximal to minimal variation of turbidity (Δ turbidity) varied with the experimental conditions. In particular, an increase in the concentration of fibrinogen was strongly associated with an increase of Δ turbidity (Fig. S1).

### Linear fit of the NT curves

We determined the slow lysis regime by fitting the NT curves between two time points corresponding to NT values of 0.01 and 0.075. Note that those two values did not define the slow regime but were only used as boundaries for the linear fit of the NT curve and chosen so that they can span a sufficiently large time window while remaining in the slow lysis regime. Linear fitting was done using the optimization and root finding from scipy.optimize. Then, to determine the ending point of the linear regime, we browsed backward along the NT curve and identified the time point on the NT curve that was distant from the linear fit by an arbitrary threshold value Δ = 0.005 (Fig. S2).

### Mathematical models of clot formation and clot lysis

The two models of clot formation and clot lysis are described in detail in the main text (see Results and discussion). Briefly, the model of clot formation consists of a set of differential equations describing the conversion of fibrinogen to fibrin monomer, the assembly of fibrin monomers into oligomers, and the association of protofibrils to form fibrin fibers. The range of values and the definitions of the kinetic parameters we used are given in Table S1, and our strategy to determine how their values were selected is described in the next section. A feature of the model is that during the formation of fibers, PAI-1 can already inactivate tPA, and Pg and tPA can reversibly bind on the fibers. The kinetic parameters for these reactions are given in Table 2. After the formation of the clot, the binding and the activation of the proteins continue (with the same parameters as in Table 2), and the lysis starts. The instantaneous rate of lysis takes into account the solubilization of fibrin (solubilization factor *γ* = 0.1) and is described by a Michaelis-Menten equation. For simplicity, we separated the formation of the clot from its lysis and assumed that the lysis started once the clot was formed. Nonetheless the two models work together as the model of fibrin polymerization provided inputs (initial conditions of bound proteins tPA^*b*^, Pg^*b*^, and fiber radius *R*_*f*0_) to the model of fibrinolysis.

**Table 2:** Simulation parameters for the binding and activation of proteins

	Kinetic parameters	Values	Units	References
Reversible binding				
Pg	$k_{f}^{Pg}$	1.087 × 10^−4^	*μ*M^−1^ s^−1^	(25,26)
	$k_{r}^{Pg}$	5.435 × 10^−5^	s^−1^	(25,26)
tPA	$k_{f}^{tPA}$	1.148 × 10^−4^	*μ*M^−1^ s^−1^	(25,27)
	$k_{r}^{tPA}$	6.658 × 10^−5^	s^−1^	(25,27)
Activation of bound species				
Pg^a^ and tPA^a^	$k_{tPA-Pg}^{2}$	0.2	s^−1^	(25,28)
	$K_{tPA-Pg}^{M}$	0.02	*μ*M	(25,28)
Plasmin^a^	$k_{Pn}^{2}$	25	s^−1^	(25,29)
	$K_{Pn}^{M}$	250	*μ*M	(25,29)
	$k_{r}^{Pn}$	5.435 × 10^−5^	s^−1^	(25,26)
Inactivation				
tPA and PAI-1	$k_{i}^{tPA-PAI}$	25	*μ*M^−1^ s^−1^	(16,30)

References cite both the works we used to get the parameter values as well as the original works in which those values were estimated.

a Indicates bound proteins.

### Choosing the kinetics parameters for the model of clot formation

From the values given in Table S1, we had more than 31,000 sets of kinetics parameters that had to be evaluated. Among them, we identified 92 that allowed us to reproduce fiber diameters consistent with those measured experimentally from electron microscopy images (see Mathematical model of thrombosis). Note that the number of sets we have identified depends on the discretization of the parameter space. The marginal probability of the parameters is presented in Fig. S3. To select the set parameters that we will use in our simulations, we proceeded as follows:

1) For each experimental condition given in Table 1, we ran the model of fibrinolysis for all the 92 sets of parameters.
2) For each simulated experiment, we stored the maximal error between experimental and simulated lysis profiles up to the total lysis time. We have chosen the maximal error to be more conservative compared to the mean error. Note that in the context of lysis, it is not possible that the simulated lysis curve deviates at a single point and then returns afterward close to the experimental lysis curve. Indeed, a deviation of a point (above the experimental curve) means that, at this point, a lot of fibrin had been lysed, but to return near the experimental curve, it would require new fibrin to be created. For similar reasons, a point cannot experience a sudden drop below the experimental curve.
3) We sorted by increasing values the stored error (each error corresponded to a set of parameters) and then scored each set of parameters by summing its rank over all experiments.
4) We then selected the set of parameters that had the smallest score. We have chosen a rank-based score to more strongly penalize the simulations with the highest error, no matter the value of the errors.

Considering the 92 sets of parameters and the 74 experiments, the worst possible score turned out to be 6808, assuming that always the same set of parameters was ranked last. We normalized the rank-based score by this value and found that our best solution had a score of 0.22. It means that, on average, this set of parameters was in the best quarter. We also found other possible candidates; in particular, there were in total 19 set of parameters that were on average in a third of the best solutions (Fig. S4 *A*). The parameters we retained for the clot formation are given in Table S1.

## Results and discussion

### Analysis of fibrin clot formation and lysis times

Fibrinogen is the key component involved in the blood clotting system. In the presence of thrombin, fibrinogen is converted to fibrin monomers after successive cleavages of the fibrinopeptides A and B. Fibrin monomers then self-assemble and polymerize into oligomers that lengthen to form protofibrils and eventually associate to build fibers. Ultimately, these fibers branch to form a three-dimensional network, which constitutes the clot.

Fibrin fibers are the main substrate for the bindings of the proteins of the fibrinolytic system. Without fibrin, affinity between tPA and Pg is extremely low and catalytic activation of Pg is inefficient. However, both the affinity and the efficiency significantly increase in the presence of fibrin, hence favoring the formation of plasmin from the conversion of Pg by tPA. On the other hand, fibrinolysis is downregulated by first, the inhibition of tPA due to the plasminogen activator inhibitor type 1 (PAI-1), and second, the inhibition of plasmin by the *α*2-antiplasmin. We analyzed clotting and fibrinolysis using the experimental procedure previously developed in (21) (see Materials and methods and Supporting materials and methods), a semiautomatic method that allows one to monitor the dynamics of fibrin assembly and dissolution from turbidity measurements. The actual measurement is the intensity of the light through the system, and high turbidity is to be understood as low attenuation or low opacity. A typical readout is presented in Fig. 1 *A*, showing the entire time course of an experiment from the formation of the clot (decrease of the turbidity) to the dissolution of the fibers (increase of the turbidity). Each step could be identified from the turbidity curve; the clot formation time (CFT) corresponded to the time needed to reach the minimum of turbidity, and the total clot lysis time (TLT) was defined as the time interval from the end of the clot formation to the point at which the derivative of the turbidity curve reaches its maximal value (fastest lysis rate). It is worth mentioning that the TLT and the end of the lysis (at which the NT returns to a value close to 1) are different. It is common not to consider as lysis time the time for complete lysis, but rather the time corresponding to the midpoint of the reduction of 50% decrease of turbidity (31, 32, 33), the time for 50% of lysis (25), or the time corresponding to the maximum of the derivative of the lysis curve (21). In all our analysis, we relied on the latter to define the lysis time. In Fig. 1 *A*, the curve was normalized to allow comparison between the different experimental conditions (see Materials and methods).

### Experimental data

Across all our experimental conditions, we measured a mean CFT of 19 min, but the values are broadly distributed (from 7 to 77 min; Fig. 1 *B*), thus indicating that the experimental conditions affect the formation time of the clot. We measured a mean TLT of 140 min also with a high dispersion (from 12 to 472 min). An important observation was that the lysis part of the curve appeared to be composed of two regimes. The first one was characterized by a slow increase of turbidity, a slow lysis regime. The second regime corresponded to a fast increase of turbidity (fast lysis regime). Independently of the experimental conditions, the slow lysis regime appeared to be a linear function of time, whereas in the fast lysis regime, the turbidity increases much faster (Fig. S5). It is thus worth discriminating those two processes in the measurement of the TLT. The average duration of the slow lysis regime was around 73 min (from 2 to 238 min). The linear slow lysis regime is characterized by its slope, and we found that the inverse of this slope was proportional to the duration of the slow lysis regime (Fig. S6 *A*). It is worth mentioning that a priori, the slope of the linear regime and its duration have no reason to be correlated. However, because the data were normalized, this correlation implied that the slow lysis regime might end for a specific value of the NT, as shown by the peak around 0.1 in the distribution of the values NT(*t* = SLT). This suggests that the duration of the slow lysis regime may be an intrinsic property (Fig. S6 *B*). The fast lysis regime was on average of 67 min (from 19 to 280 min), and for the definition of total lysis used here, the slow lysis regime accounted for a significantly longer duration than the fast lysis regime (Fig. 1 *C*). Fig. S6 *A* shows that if the slow lysis regime is long, it will also be the case for the fast lysis regime. From a practical point of view, we show that by measuring the slope of the turbidity curve in the slow lysis regime, it becomes possible to provide an estimation of the TLT from early measurements (waiting half the time, on average) without waiting until the actual end of the lysis.

**Figure 2:**
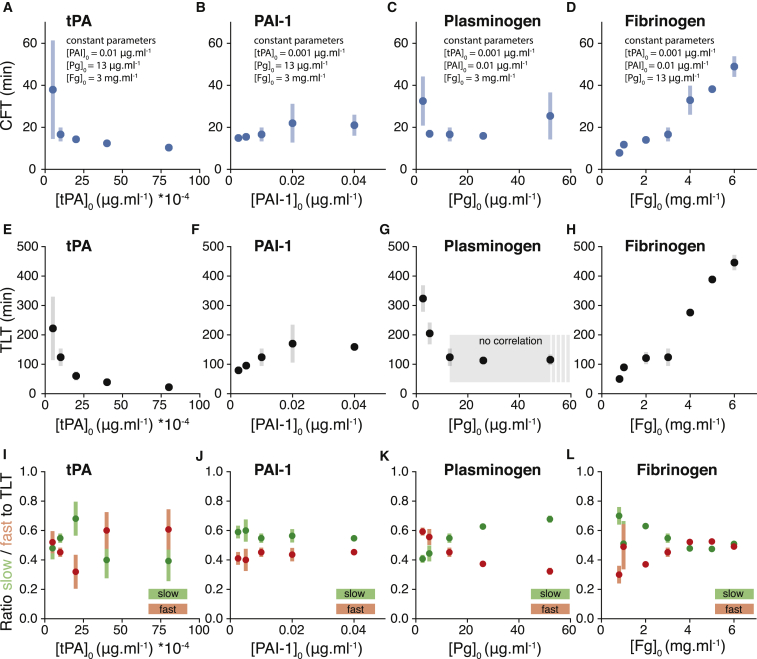
Clot formation and lysis times for different experimental conditions. (*A*–*D*) Mean CFTs. (*E*–*H*) Mean TLTs. (*I*–*L*) Mean ratio of slow (*R*_*SL*_, *green*) and fast (*R*_*FL*_, *red*) times with respect to the total lysis time. Bars represent the standard deviation. Only one experimental condition was varied at a time, and all the others remained constant (the constant parameters are listed on the *top row* and are the same used in the corresponding column). To see this figure in color, go online.

We then investigated how each event depends on the initial experimental conditions. We compared the CFT for different concentrations of tPA, PAI-1, Pg, and fibrinogen (Fig. 2*, A–D*). As expected, we found that the initial concentration of tPA has a strong effect on the CFT, which decreases as the tPA concentration increases. An explanation is that an excess of tPA rapidly increases plasmin production in the presence of a small amount of fibrin, and it is known that plasmin can induce the production of a clottable form of fibrinogen (34), thus reducing the time to form the clot. Conversely, the CFT significantly increased with PAI-1 (Spearman correlation *ρ*_*s*_ = 0.44, *p*_*val*_ = 0.016), indicating that PAI-1 inhibited tPA before lysis. Considering the time required to form the clot and the fast kinetics of inhibition of tPA by PAI-1 (second-order rate constant $k_{i}^{tPA-PAI}$ on the order of 10^7^ M^−1^ s^−1^ (35)), tPA might bind early during the polymerization of fibrin and the formation of the fibers. If not, most of the initial tPA would be already inhibited, and no lysis could be observed. We found no correlation between the CFT and the concentration of Pg. Instead, the CFT correlated with the initial concentration of fibrinogen mainly because there was more material available to build the clot, as shown by the variation of turbidity Δ*T* between the beginning of the experiment and the end of the clotting (Fig. S1 *D*). To a lesser extent, Δ*T* decreased (resp. increased) with tPA (resp. PAI-1) jointly with the time needed to form the clot.

Then, we investigated the dynamics of the fibrinolysis. As expected from the respective roles of the different components of the fibrinolytic system, the TLT increased with the concentrations of PAI-1 and fibrinogen (Fig. 2*, F and H*), and it decreased with the concentration of tPA and Pg. However, the effect of Pg was mainly significant for concentrations lower than 13 *μ*g ⋅ mL^−1^. Above this value, the TLT saturated, suggesting that we covered a sufficiently large range of values for Pg, even with concentrations lower than physiological ones. Comparing the slow and the fast lysis regimes side by side, we could observe how the initial conditions affect the relative contribution of each regime on the TLT. For each experiment, we measured the ratios *R*_*SL*_ = $\frac{SL}{SL+FL}$ and *R*_*FL*_ = $\frac{FL}{SL+FL}$, and we averaged them over identical experimental conditions. When one of the two ratios is greater than 0.5, the corresponding regime contributes more to the TLT than the other.

The relative contributions of the slow and fast lysis regimes did not display a very strong association with the different values of [tPA]_0_. Both *R*_*SL*_ and *R*_*FL*_ were fluctuating around 0.5 (Fig. 2 *I*), thus suggesting that the relative durations of the slow and fast lysis regimes were similar for the range of tPA concentrations we used in our experiments. More precisely, we observed that the fast lysis times rapidly decreased with the concentration of tPA and eventually plateaued for [tPA]_0_ ≥ 0.002. On the other hand, the slow lysis time decreased less rapidly and became similar to the fast lysis time for [tPA]_0_ ≥ 0.004 (Fig. S7 *A*). Regarding the effect of PAI-1, both slow and fast lysis times increased with the values of [*PAI*-1]_0_ (Fig. S7 *B*), and for any values of [*PAI*-1]_0_, the slow lysis regime dominated the fibrinolysis time (Fig. 2 *J*). For higher concentration of tPA, we observed a similar behavior for the slow lysis time but a saturation of the fast lysis times (Fig. S7 *C*), thus indicating that at a physiological concentration of tPA, the total lysis time was modulated by the slow lysis regime rather than the fast lysis regime. Pg and fibrinogen displayed opposite trends (Fig. 2*, K and L*). Increasing [Pg]_0_ corresponded to a decrease of *R*_*FL*_, with a crossing point around [Pg]_0_ = 13 *μ*g ⋅ mL^-1^. This crossover was due to a saturation of the slow lysis times, whereas the fast lysis times kept decreasing with an increase of Pg (Fig. S7 *D*). An increase of [*Fg*]_0_ corresponded to an increase of *R*_*FL*_, with the two curves collapsing around [*Fg*]_0_ = 4 mg ⋅ mL^-1^. The relative contribution may depend on the definition of the lysis time. Nonetheless, regardless of the definition considered, our results suggest that the lysis is in the slow regime over a significant amount of time (Fig. S8). It is worth mentioning that in this study, we neither investigated experimentally the effect of the plasmin inhibitor *α*2-antiplasmin nor the impact of thrombin-activatable fibrinolysis inhibitor on the lysis time. However, the linear regime was also observed in an euglobulin clot lysis assay (18), thus indicating that the linear regime also holds in more complex situation.

### Modeling clot formation and fibrinolysis

Our analysis of turbidity curves revealed two features of the clot formation and lysis process that are important for the development of the models. First, the binding of proteins has to be considered during the assembly of the clot, and second, a significant part of the lysis appeared to be inefficient. For the sake of simplicity, we decided to decouple the formation of the clot from its lysis, and we used two different models to describe our experiments. That is to say, we made the assumption that the lysis started once the clot was formed. We adapted a kinetic model of fibrin polymerization from (36) to estimate the concentrations of bound species after clotting, and we modified the fibrinolysis model from (25) to describe the slow and fast lysis regimes. In general, theoretical approaches consider either kinetic reactions at the surface of the fibers or in the volume of the fibers. In our case, we hypothesized that the slow and fast regimes are the result of a transition between surface and bulk binding of the proteins.

#### Mathematical model of thrombosis

**Figure 3:**
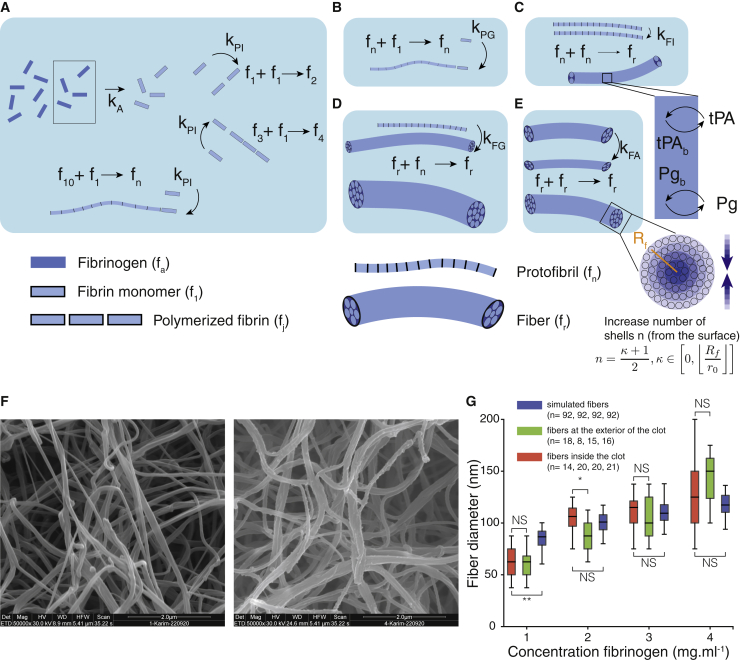
Graphical representation of the model of clot formation. (*A*) Conversion of fibrinogen to fibrin and polymerization of fibrin monomers until the threshold size is reached to form protofibril. (*B*) Protofibrils grow in length by addition of fibrin monomers. (*C*) Association of protofibrils to form fibers. Binding of tPA and Pg starts when fibers are created. (*D*) Fibers grow by addition of protofibrils. (*E*) Two fibers aggregate together; a transverse view of the fibers shows the radius of the fiber (*R*_*f*_) and the increasing number of shells from the surface to the center. In this example, for the bulk reaction (i.e., in the entire fiber), we have *n* = 5 and *κ* = 9. (*F*) Scanning electron microscopy images of fibrin fibers at fibrinogen concentration of 1 mg ⋅ mL^−1^ (*left*) and 4 mg ⋅ mL^−1^ (*right*). The scale bar represents 2 *μ*m. (*G*) Diameter of the fibers for different concentrations of fibrinogen measured in the interior of the clot (*red*), at the exterior of the clot (*green*) and for simulated fibers (*blue*). The boxplots represent the median as well as first and third quartiles. ∗∗*p* < 0.01; ∗*p* < 0.05; NS, not significant. To see this figure in color, go online.

The formation of a fibrin clot is a stepwise process resulting from the conversion of fibrinogen into fibrin by the cleavage of fibrinopeptide A by thrombin, the polymerization of fibrin monomers to protofibrils, and the aggregation of protofibrils to form fibers that eventually merge and branch to create a mesh of fibers (Fig. 3*, A–E*; Table S1). In (36), the authors proposed that protofibrils must reach a minimal length before aggregation to reproduce the lag period observed in turbidity experiments. We chose a length of 11 monomers as in (36). Note that different threshold lengths can be found in the literature, but the values remained similar. In (37), the authors used a threshold of 12 monomers. The equations of the kinetics of the fiber aggregation are the following:

(1) $$\begin{array}{l} \frac{df_{a}}{dt}=-k_{A}f_{a}\\ \frac{df_{1}}{dt}=k_{A}f_{a}-k_{PI}f_{1}\left(f_{1}+\sum_{j=1}^{10}f_{j}\right)-k_{PG}f_{1}f_{n}\\ \frac{df_{j}}{dt}=k_{PI}f_{1}\left(f_{j-1}-f_{j}\right)\\ \frac{df_{n}}{dt}=k_{PI}f_{1}f_{10}-2k_{FI}f_{n}f_{n}-k_{FG}f_{r}f_{n}\\ \frac{df_{r}}{dt}=k_{FI}f_{n}f_{n}-k_{FA}f_{r}f_{r}, \end{array}$$

where *f*_*a*_ is the fibrinogen, *f*_*j*_ the fibrin oligomers of size *j*, *f*_*n*_ the protofibrils, and *f*_*r*_ represents the fibers; the summation $\sum_{j=1}^{10}$ accounts for the minimal length before aggregation. The total amount of protofibrils in fibers $f_{n}^{tot}$, the total amount of fibrin in protofibrils *C*_*fn*_, and the total amount of fibrin in fibers *C*_*fr*_ are given by

(2) $$\begin{array}{l} \frac{df_{n}^{tot}}{dt}=2k_{FI}f_{n}f_{n}+k_{FG}f_{r}f_{n}+k_{FA}f_{r}f_{r}\\ \frac{dC_{fn}}{dt}=11k_{PI}f_{1}f_{10}+k_{PG}f_{1}f_{n}-2k_{FI}f_{n}C_{fn}-k_{FG}f_{r}C_{fn}\\ \frac{dC_{fr}}{dt}=2k_{FI}f_{n}C_{fn}+k_{FG}f_{r}C_{fn}. \end{array}$$

The model described above proposes an oversimplified situation of real clot formation. Yet, it remains quite complex and relies on kinetic parameters that are largely unknown. This model assumes that protofibrils have to grow before aggregation, but we only considered addition of one monomer to an existing protofibril and neglected all possible permutations of oligomers of different size. Weisel et al. modeled some of these oligomer-oligomer interactions but reported small differences in their results (36). Additionally, in (38) the authors mentioned that these interactions were unlikely to occur except for oligomers of size two. Nonetheless, accounting for those interactions would increase drastically the computational cost and the complexity of the model. It was also assumed that fibrinogen is directly converted into polymerizable fibrin monomer without accounting for the formation of the fibrinogen-thrombin complex and the sequential release of fibrinopeptides A and B. Another simplification of the model was that it considered only lateral aggregation and did not account for the branched structure of the fiber network. The three-dimensional structure and stability of the network are known to have an impact on the rheological and mechanical properties of the clot as well as on the plasmin-catalyzed fibrinolysis. Whereas more detailed molecular models (39,40) could predict with greater details fiber properties during network formation, they operate at space and timescales (nanometers and microseconds) that are not compatible with our in vitro experiments (typically hundreds of seconds for centimetric clots). Despite the aforementioned simplifications and the lack of knowledge of the kinetic parameters of clotting, the model of Weisel et al. (36) remains a good compromise between molecular and molar levels of description and could be easily extended to determine the number of binding sites available for reactions during clot formation.

Knowing the total number of protofibrils in the fibers and the number of fibers, the average fiber radius *R*_*f*_ is given by

(3) $$R_{f}=\sqrt{\frac{f_{n}^{tot}}{f_{r}}\frac{1}{\pi p_{0}}},$$

where *p*_0_ = 0.01116 protofibrils/nm^2^ is the density of protofibrils in the fiber (41). Using the radius *r*_0_ of a protofibril, Diamond et al. calculated the number of protofibrils in *κ* layers of a fiber of radius *R*_*f*_ (25):

(4) $$m\left(R_{f}\right)=\int_{0}^{2\pi }\int_{R_{f}-\kappa r_{0}}^{R_{f}+r_{0}}p_{0}\;r\;dr\;d\theta =\pi p_{0}\left(2r_{0}R_{f}\left(1+\kappa \right)+r_{0}^{2}\left(1-\kappa^{2}\right)\right).$$

For *κ* = 0, reactions occur at the very surface of the fibers (i.e., only the exposed side of the outer protofibrils), whereas for *κ* = $\frac{R_{f}}{r_{0}}$, reactions occur in the entire volume of the fibers. Using Eq. 2, we calculated the number of fibrin monomers per protofibril and, using Eq. 4, the number of fibrin monomers on the outer part of the fibers, thus obtaining the number Θ of sites on the fiber that are available for binding:

(5) $${\text{Θ}}^{tPA,Pg}\left(R_{f}\right)=\frac{C_{fr}}{f_{n}^{tot}}\times m\left(R_{f}\right)\times q_{tPA,Pg},$$

where *q* is the number of binding sites per monomer of fibrin for tPA (*q*_tPA_ = 1.5) and Pg (*q*_Pg_ = 2.4) (25). We will discuss later how this number of shells plays a role in the dynamics of the lysis. However, during the formation of the fibers, we only considered adsorption of the proteins. Knowing the number of binding sites on the fibers during the formation of the clot, we could estimate the amount of bound and free proteins at the end of the clotting process. Binding was described with second-order rate equations, unbinding with first-order rate equations, and the Michaelis-Menten equation was used for the formation of bound plasmin (*Pn*^*b*^) by the activation of Pg by tPA^*b*^. The time evolution of these proteins in their free and bound forms (tPA^*b*^, Pg^*b*^, and Pn^*b*^) were determined with the following set of equations, using the parameters given in Table 2:

(6) $$\begin{array}{l} \frac{d\left[tPA\right]}{dt}=-k_{f}^{tPA}\left[tPA\right]\left({\text{Θ}}^{tPA}-\left[tPA^{b}\right]\right)+k_{r}^{tPA}\left[tPA^{b}\right]-k_{i}^{tPA-PAI}\left[tPA\right]\left[PAI-1\right]\\ \frac{d\left[tPA^{b}\right]}{dt}=k_{f}^{tPA}\left[tPA\right]\left({\text{Θ}}^{tPA}-\left[tPA^{b}\right]\right)-k_{r}^{tPA}\left[tPA^{b}\right]\\ \frac{d\left[Pg\right]}{dt}=-k_{f}^{Pg}\left[Pg\right]\left({\text{Θ}}^{Pg}-\left[Pg^{b}\right]-\left[Pn^{b}\right]\right)+k_{r}^{Pg}\left[Pg^{b}\right]\\ \frac{d\left[Pg^{b}\right]}{dt}=k_{f}^{Pg}\left[Pg\right]\left({\text{Θ}}^{Pg}-\left[Pg^{b}\right]-\left[Pn^{b}\right]\right)-k_{r}^{Pg}\left[Pg^{b}\right]-k_{tPA-Pg}^{2}\frac{\left[tPA^{b}\right]\left[Pg^{b}\right]}{K_{tPA-Pg}^{M}+\left[Pg^{b}\right]}\\ \frac{d\left[PAI-1\right]}{dt}=-k_{i}^{tPA-PAI}\left[tPA\right]\left[PAI-1\right]\\ \frac{d\left[Pn^{b}\right]}{dt}=-k_{r}^{Pn}\left[Pn^{b}\right]+k_{tPA-Pg}^{2}\frac{\left[tPA^{b}\right]\left[Pg^{b}\right]}{K_{tPA-Pg}^{M}+\left[Pg^{b}\right]}. \end{array}$$

Here, we did not account for the direct conversion of Pg by tPA in the free phase because it is kinetically slow compared to the formation of bound plasmin from tPA^*b*^ and Pg^*b*^ (between two and three orders of magnitude (16,25)). For the sake of simplicity, we did not consider a potential early lysis simultaneously with the formation of the fibers, but it remains possible that the lysis started before the complete formation of the clot, in particular for the clot at a high concentration of fibrinogen.

Because the experiments were conducted without flow and the different species were homogenized at the beginning of the experiments, we did not incorporate advection or diffusion in the model. The role of the diffusion is discussed elsewhere (37,38) in the context of sol-gel phase transition. The role of the clot formation model was to generate input conditions: concentrations of tPA^*b*^ and Pg^*b*^, and the initial fiber radius *R*_*f*0_ for the lysis model. As already discussed, without binding occurring during the formation of the clot, no lysis would be possible (at least with our experimental conditions) because of the fast inhibition of tPA by PAI-1.

The experimental values of the kinetic parameters of the polymerization remain largely unknown despite recent experimental progress in live observations of fibrin polymerization (42). We performed a variance-based sensitivity analysis to investigate the effect of these parameters on the concentration of tPA^*b*^ and *R*_*f*0_. We computed the first-order, second-order, and total-order Sobol indices with parameters sampled with the Saltelli scheme (43) (Fig. S9*, A and B*) from the values given in Table S1. For this analysis, all sampled parameters were considered. We did not use any constraints on the diameter of the fibers, as opposed to the comparison we made previously with electron microscopy images to choose the kinetic parameters for the clot formation model (see Materials and methods). For the concentration of tPA^*b*^, we found that the most influential parameters were first *k*_*FG*_ (representing the addition of protofibrils to growing fibers) and *k*_*FA*_ (describing the aggregation of fibers), then *k*_*PG*_ (growth in length of protofibrils) and *k*_*A*_ (cleavage of fibrinopeptides A). On the other hand, *R*_*f*0_ was influenced by *k*_*FG*_, *k*_*A*_, *k*_*FA*_, and *k*_*PG*_ (Fig. S9 *D*). We then ran the model of clot formation for all possible sets of parameters (Table S1), which represents more than 31,000 simulations for each concentration of fibrinogen between 1 and 4 mg ⋅ mL^−1^. We used the initial concentrations of [tPA]_0_ = 0.001 *μ*g ⋅ mL^−1^, [Pg]_0_ = 13 *μ*g ⋅ mL^−1^, [*PAI*-1]_0_ = 0.01 *μ*g ⋅ mL^−1^, and we set the clotting time to 1000 s (which was similar to the average CFT). In parallel, we measured fiber diameters from electron microscopy images of clot made in similar experimental conditions (Fig. 3 *F*), and we retained the kinetics parameters that provide similar fiber diameters (Fig. 3 *G*), assuming that at least 80% of the initial fibrinogen was incorporated to the fibers. Among the 31,000 sets, we identified 92 that reproduced the fiber diameters measured experimentally. There were no statistical differences between simulated fiber diameters and fiber diameters measured inside the clot, except at low concentration of fibrinogen, most likely because the simulation time was too long for these conditions. We observed that the diameters of the fibers at the exterior of the clot seemed to be more dependent on the concentration of fibrinogen compared to fibers inside the clot, but for endogenous lysis, we were rather interested in the fibers inside. Following the procedure described in the Choosing the kinetics parameters for the model of clot formation, we found that the set with the values *k*_*A*_ = 0.02 s^−1^, *k*_*PI*_ = 10^−17^$\frac{L}{molecules\cdot s}$, *k*_*PG*_ = 10^15^$\frac{L}{molecules\cdot s}$, *k*_*FI*_ = 10^−18^$\frac{L}{molecules\cdot s}$, *k*_*FG*_ = 10^−16^$\frac{L}{molecules\cdot s}$, and *k*_*FA*_ = 10^−18^$\frac{L}{molecules\cdot s}$ had the best ranked score (Table 1; Fig. S4). Although the best and worst sets of parameters have similar *R*_*f*0_, it is interesting to note that they have distinct concentrations of tPA^*b*^ (Fig. S10*, A and B*). The best and worst sets of parameters had identical parameters except for *k*_*FI*_ and *k*_*A*_, whose values were, respectively, 10^−19^$\frac{L}{molecules\cdot s}$ and 0.002 s^−1^. From the best-ranked set of parameters, we found that the model of clot formation resulted in a concentration of tPA^*b*^ around 2% of the initial concentration of tPA.

#### Mathematical model of fibrinolysis

To model the plasmin-mediated lysis of the fibrin clot, we adapted the framework proposed in (25) and more recently used in (44, 45, 46). Briefly, the authors proposed that the lysis proceeds from the outside of the fibers, thus reducing their diameter and eventually leading to the lysis of the clot. It is known that plasmin cuts the fibers transversely, but it was not clear whether the fibers were ultimately transected or radially lysed because of the uniform distribution of cleavage sites along the fibers. There is now evidence in favor of the first mechanism, both experimentally (47,48) and theoretically (49,50). However, Piebalgs et al. reported that their results for a one-dimensional diffusion model were very similar (in term of lysis front velocities) to those obtained in (49). In (45), the authors draw the conclusion that the similarity between their results and those obtained in (49) confirmed the relevancy of using the approach of Diamond of a fibrin microstructure model for macroscopic simulations of clot lysis, despite the assumption of homogeneous shrinkage of the fibers. Knowing the amount of bound plasmin [*Pn*]_*b*_, it was thus possible to estimate the historic amount of lysis *L*(*t*) by integrating over time the instantaneous rate of lysis kinetically described by a Michaelis-Menten equation:

(7) $$L\left(t\right)=\gamma \int_{0}^{t}\frac{k_{Pn}^{2}\left[Pn^{b}\right]{\text{Θ}}^{Pn}\left(\tau \right)}{K_{Pn}^{M}+{\text{Θ}}^{Pn}\left(\tau \right)}d\tau ,$$

where *γ* = 0.1 is the solubilization factor, i.e., the quantity of fibrin that returns to the plasma phase per number of plasmin cleavage. The percentage of lysis was then obtained by dividing the historical amount of lysis *L*(*t*) by the initial amount of fibrin. Because the lysis proceeds from the outside shell, *R*_*f*_ is directly proportional to the amount of lysis *L*(*t*) (25):

(8) $$R_{f}^{2}\left(t\right)=R_{f0}^{2}-\left(\frac{1}{\pi p_{0}}\right)\frac{L\left(t\right)}{\left(\frac{L_{f}}{V_{c}}\right)\lambda_{f}\frac{1}{N_{av}}},$$

where *L*_*f*_, the average length of the fibers, remains constant during the lysis and was estimated from geometrical and conservation considerations. *V*_*c*_ is the volume of the clot (which was assumed to be conserved during the lysis, whereas the volume of the fibers changed), *N*_*av*_ is the Avogadro number, and *λ*_*f*_ is the linear density of fibrin monomers along the fibers. Simultaneously with the decrease of *R*_*f*_, the clot porosity *ϵ* also varies as

(9) $$\varepsilon =1-R_{f}^{2}\left(t\right)\frac{L_{f}}{V_{c}}.$$

It is important to note that for the fibrinolysis, the bound concentration is defined with respect to fibrin phase volume. For any species *i* with an initial concentration in the fluid phase [*i*]_0_, the mass conservation implies that [*i*] + $\frac{1-\varepsilon }{\varepsilon }$[*i*]^*b*^ = [*i*]_0_. The rates of change for the species in the fluid phase are

(10) $$\begin{array}{l} \frac{d\left[tPA\right]}{dt}=-\left(\frac{1-\varepsilon }{\varepsilon }\right)\frac{d\left[tPA^{b}\right]}{dt}-k_{i}^{tPA-PAI}\left[tPA\right]\left[PAI-1\right]\\ \frac{d\left[Pn\right]}{dt}=-\left(\frac{1-\varepsilon }{\varepsilon }\right)\frac{d\left[Pn^{b}\right]}{dt}\\ \frac{d\left[Pg\right]}{dt}=-\left(\frac{1-\varepsilon }{\varepsilon }\right)\frac{d\left[Pg^{b}\right]}{dt}. \end{array}$$

In (25), the authors mainly consider reactions at the outer shell of the fibers but also compare the time to achieve 50% average lysis with different numbers of shells. Nonetheless, with or without the steric constraint, they kept the number of shells constant (between 1 and 50 shells) during their simulations. Instead, we hypothesized that this number is changing dynamically during the lysis, thus resulting in the different regimes observed in our experiments.

**Figure 4:**
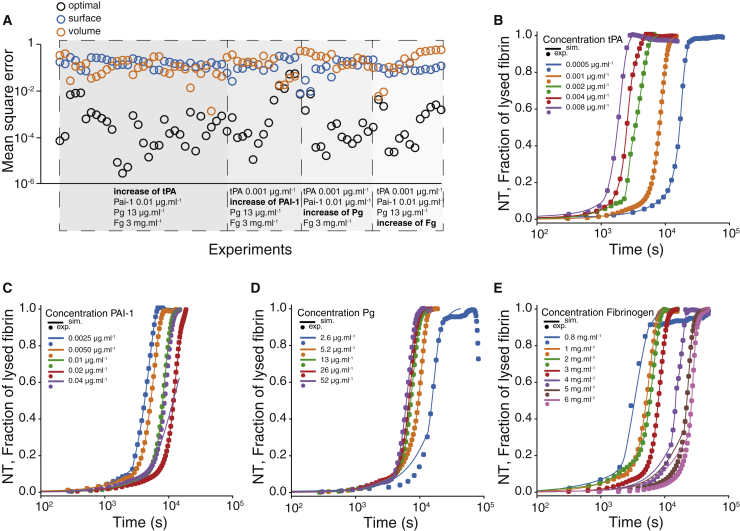
Comparison between simulated and experimental lysis profiles. (*A*) Time average of the squared error (squared distance between simulated and corresponding experimental lysis profiles) for each simulation with optimal number of shells (*black empty circles*), with the shell only at the surface of the fibers (*empty blue circles*), and with all available shells in the entire fibers (*empty orange circles*). In each gray box, one concentration was varied (in *bold*, with an increase from left to right along the *x* axis) and all other concentrations stayed constant. Time courses of NT and fraction of lysed fibrin for different initial concentrations of (*B*) tPA, (*C*) PAI-1, (*D*) plasminogen (Pg), and (*E*) fibrinogen are shown. All simulations were performed with the parameters given in Table S1. To see this figure in color, go online.

We did not model how the number of shells varied during the lysis; rather, we estimated at each time step the optimal value that best fit our experimental turbidity curves, based on the fact that NT is a good proxy for the concentration of fibrin in the fibers. Yet, we will see that this approach provides relevant information for the elaboration of a surrogate model that does not require dynamical optimization. In practice, for each experimental condition, we first interpolated linearly the turbidity curves between the experimental time points to achieve a similar resolution in time as in simulations (Δ*t* = 0.1 s). Then, we ran the model of clot formation to obtain the initial conditions for the lysis (concentrations of bound and free proteins, amount of fibrin incorporated to the fibers, and *R*_*f*0_). During the simulation of the lysis, we had to estimate at each time *t* the number of shells that are used for the reactions. At each time step, we determined this number by solving independently all the equations of fibrinolysis for all the values of the number of available shells. The optimal number of shells was defined as the value that, at a time *t*, minimized the squared error between *L*^*sim*^(*t* + Δ*t*), the simulated amount of lysis at the next iteration, and the experimental one, given by NT(*t* + Δ*t*). Once this value was obtained, the equations for the fibrinolysis were actually computed for the time *t* + Δ*t* and the simulation continued by repeating the optimization procedure until all fibrin was consumed. It is worth mentioning that the optimal number of shells could not be freely chosen because it was constrained by the instantaneous number of available shells; hence, the difference between *L*^*sim*^(*t* + Δ*t*) and NT(*t* + Δ*t*) could not be made arbitrarily small. Considering that the state of the system at time *t* depended on all the history of the lysis, errors could potentially accumulate during the simulations despite the optimization procedure. Overall, we found that the mean-square error between the simulated and experimental lysis curves stayed quite small for most of the experimental conditions. Importantly, our model appeared to be able to better perform compared to the standard approaches with kinetic reactions only at the surface or in the volume of the entire fiber (Fig. 4 *A*). Both have higher errors compared to the model with the time-changing number of shells. This supports the idea that a transition between surface and bulk reactions may be a relevant mechanism regarding the transition between slow and fast lysis, as a surface-only or volume-only reaction model could not capture it.

As a further investigation of the lysis model, we directly compared the simulated lysis profiles with the corresponding experimental one. Our results are summarized in Fig. 4*, B–E*. The change in the experimental conditions is indicated on each figure, and all other concentrations stay the same. Overall, we found a very good agreement between the experiments and the simulations, both being indistinguishable most of the time. In particular, the results for the change of tPA matched the experiments almost perfectly (Fig. 4 *B*). Note, however, that our model poorly captured the experimental fast lysis regime for the highest concentration of PAI-1 (Fig. 4 *C*). Numerical simulations predicted a long TLT, as one would expect considering that PAI-1 inhibits tPA. Yet, the TLT was experimentally very short (Fig. 2 *F*). We could not exclude an experimental issue in this case; two out of the four replicates with the highest concentration of PAI-1 had short TLTs, whereas the two other replicates did not have any lysis at all, thus indicating that experiments with too high a concentration of PAI-1 were hard to conduct and that the results are not totally reliable.

The slow lysis regime was difficult to recapitulate for the lowest concentration of Pg, but overall, we also found a good agreement with the experimental curves (Fig. 4 *D*). Another discrepancy between simulation and experiment was observed for concentrations of fibrinogen greater than 5 mg ⋅ mL^−1^ (Fig. 4 *E*). Between the end of the slow lysis regime and the start of the fast lysis regime, the model failed to reproduce the observations. The slow lysis was found in the simulation to be faster than in the experiments, yet the fast lysis regime was recovered at high fibrinogen concentration. It is likely that this difference between the simulations and the experiments was due to an earlier start of the lysis that we did not consider in the clot formation. If, at some point, clotting and lysis balance, our approach would detect the end of the clotting too early and initiate the fibrinolysis too early, too, hence shortening the slow lysis regime in the simulations.

**Figure 5:**
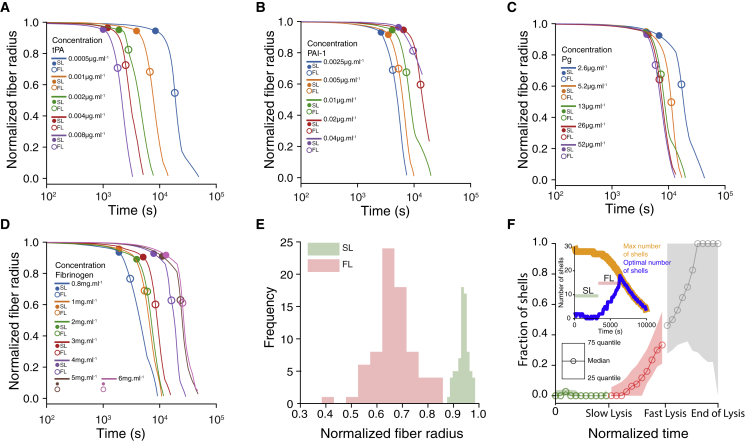
Time evolution of the normalized fiber radius ${\tilde{R}}_{f}$ for various concentrations of (*A*) tPA, (*B*) PAI-1, (*C*) Pg, and (*D*) fibrinogen. Unless otherwise noted, the concentrations are fibrinogen = 3 mg ⋅ mL^−1^, tPA = 0.001 mg ⋅ mL^−1^, Pg = 13 mg ⋅ mL^−1^, and PAI-1 = 0.01 mg ⋅ mL^−1^. (*E*) Distribution of the values of the fiber radius at the end of the slow lysis regime (*green*) and fast lysis regime (*red*). (*F*) Time evolution of the fraction of shells during the slow regime (*green*), fast regime (*red*), and from the end of the fast regime to the complete lysis (*gray*). Empty circles represent the median and the filled curves indicate the first and third quartiles. To aggregate all simulations, time was normalized as follows: each simulation was split into three regions according to their slow lysis time, fast lysis time, and complete lysis. Then, in each of these regions, the time was normalized by the length of the corresponding regime such that each region got a normalized time between 0 and 1. Each region was separated in 10 bins, and the fraction of shells was averaged in each bin. Each region was then displayed side by side to reconstruct the time evolution of the fraction of shells with normalized time. Median and quartiles were shown instead of mean and standard deviation to avoid having error bars overflowing the negative part of the *y* axis. Inset: time evolution of the optimal number of shells (*blue*) and the maximal number of available shells (*orange*). The optimal number of shells increased in time and eventually reached the maximal number of shells available. Then, it decreased as the lysis proceeds. The large values of 25–75 quartiles in the gray region (up to the complete lysis) were mainly due to the use of the fraction of shells. As the lysis proceeds, the total number of shells decreased; thus, small variations in the optimal number of shells appeared as large variation in the fraction of shells. All simulations were performed with the parameters given in Table S1. To see this figure in color, go online.

We then investigated the change in time of *R*_*f*_ (Fig. 5). As expected, *R*_*f*_ invariably decreased as the lysis proceeds. For all conditions, we observed two regimes: the first one, in which *R*_*f*_ decreased slowly (up to the corresponding experimental slow lysis time), and the second one, in which *R*_*f*_ dropped sharply to a value close to 0. More interestingly, the distributions of the normalized fiber radius ${\tilde{R}}_{f}=\frac{R_{f}}{R_{f0}}$ at the end of each regime were both nicely peaked, between 0.6 and 0.7 for the fast lysis regime and between 0.9 and 1 for the slow lysis regime. As already shown by the distribution of NT at the end of the slow lysis regime (Fig. S6 *B*), the distribution of ${\tilde{R}}_{f}$ is in agreement with the idea that the transition from slow to fast lysis regime may be an intrinsic property.

We have not yet discussed the time evolution of the number of shells during the lysis. According to our hypothesis, if there is a dynamic transition between surface and bulk reactions, this should be reflected by an increase of the number of shells as the lysis advances. To aggregate and make the different experimental conditions comparable, we first had to normalize the time so that the slow and fast lysis regimes had the same duration. For each simulation, the corresponding experimental slow lysis and fast lysis times were known, and we could also measure the end point of the lysis (marked by a value of NT = 0.95). Then, each simulation was divided into three distinct parts (SL, FL, and end of lysis), and the time was normalized by the length of each respective regime. Put another way, the slow lysis time, fast lysis time, and end of lysis time became, respectively, 1, 2, and 3. Finally, for each part with normalized time, we aggregated the fraction of shells (i.e., the number of optimal shells divided by the number of available shells) in bins of length 0.1. Using this reconstructed time evolution, we found that the fraction of shells invariably increased with time (Fig. 5 *F*) and eventually reached the maximal number of shells available in the fibers (*inset* in Fig. 5 *F*).

#### Surrogate model of fibrinolysis

**Figure 6:**
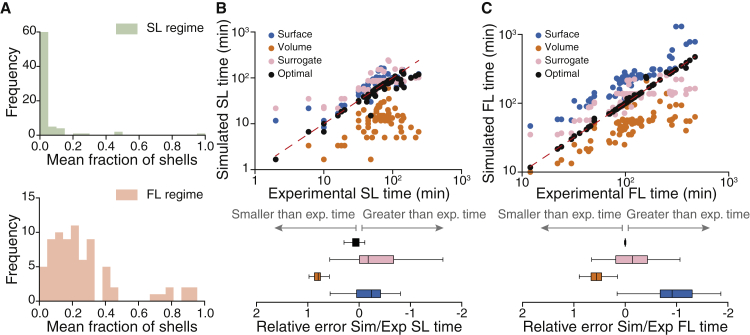
Comparison between the surrogate lysis model and the models with optimal fraction of shells, surface reactions only, and volume reactions only. (*A*) Distribution of the mean fraction of shells in the slow lysis regime (*green*, *upper panel*) and fast lysis regime (*red*, *lower panel*). Comparison between simulated and experimental slow (*B*, *upper panel*) and fast (*C*, *upper panel*) lysis times for the models with surface reactions (*blue circles*), volume reactions (*orange circles*), surrogate (*pink circles*), and optimal number of shells (*black circles*). Relative errors between the experimental and simulated times are shown in the corresponding lower panel. The boxplots represent the median as well as first and third quartiles. To see this figure in color, go online.

Finally, we combined our results to propose a surrogate model that could reasonably predict slow and fast lysis times without relying on empirical curve fitting. The underlying idea of this surrogate model is to use the distribution of the fraction of shells in the different regimes instead of searching for the optimal fraction of shells at each time step. To do so, first we need the distribution of the fraction of shells in both regimes, and second, we need to identify, without using experimental curves, the time points of the transition between the slow and fast lysis regimes. The distribution of the fraction of shells can be calculated from Fig. 5 *F*. For each simulation, we computed the mean fraction of shells in the slow and fast regimes and built the two corresponding distributions (Fig. 6 *A*, *upper panel* for the slow lysis regime and *lower panel* for the fast lysis regime). Next, we found the transition without relying on the experiments. As discussed before, it is possible to identify in which state the system is by measuring ${\tilde{R}}_{f}$. We made the simplification that if ${\tilde{R}}_{f}$ > 0.9, then the system is in the slow lysis regime; otherwise, it is in the fast lysis regime. With this surrogate model, it is possible to solve numerically the equations of the lysis without using any extra information. At each time step, ${\tilde{R}}_{f}$ was calculated to determine the state of the system; if we found ${\tilde{R}}_{f}$ > 0.9, the fraction of shells was drawn randomly from the distribution shown in the upper panel of Fig. 6 *A*; otherwise, it was drawn from the distribution shown on the lower panel. Note that this surrogate model bypasses the fitting step but still requires the equations of the lysis to be solved.

We compared the slow and fast lysis times of this surrogate model with the results of the models with optimal number of shells, surface reactions only, and bulk reactions only (Fig. 6*, B and C*). For these comparisons, the slow and fast lysis times for the surrogate model were determined according to the value of ${\tilde{R}}_{f}$ (0.9 for the slow lysis regime and 0.6 for the FL), whereas for the other models, we matched the time points at which the amount of lysed fibrin was equal to NT at the slow and fast lysis times. The surface and volume models did not display a clear change in the regime of lysis compared to experimental curves, thus making the use of the linear fit difficult. Instead, using the matched time points, we could compare the different models in a self-consistent manner. As anticipated, the model with surface reactions resulted in longer lysis times (both slow and fast lysis times) compared to the model with volume reactions (Fig. 6*, B and C*). The surface model better reproduced the slow lysis times compared to the volume model, in agreement with the hypothesis of surface reactions during the slow lysis regime, and the distribution of the relative error of slow lysis times for the surrogate model was very similar to the distribution of the relative error of the surface model (Fig. 6 *B*, *lower panel*). The estimated fast lysis times for the surface (resp. volume) models were longer (resp. shorter) compared to the experiments, whereas the times predicted by the surrogate model were between them and closer to the optimal model. The results for the surrogate model were more dispersed than for the optimal one because we lost the smooth temporal evolution of the fraction of shells by using random distributions. Nonetheless, this surrogate model predicts more reliably both the slow and fast lysis times than the standard approaches based on either surface or bulk reactions. Our model hypothesized a uniform distribution of protofibrils across the fiber. However, it has been shown that the distribution of protofibrils in the cross section of a fiber is not homogeneous. Instead, protofibrils are more densely packed in the core of the fiber than on the outer part, with a fractal lateral structure (22,51, 52, 53). We modified our model to estimate the number of binding sites on the outer shell using the distribution of protofibrils as function of the fiber radius given in (22). We found that both the latency and lysis times were similar to those obtained with the surface model, though slightly better, but not as accurate as the model with optimal number of shells and the surrogate model (Fig. S11). The median relative error for the slow regime was near zero (Fig. S11*, A and B*), but the median value for the fast regime was shifted toward negative values (Fig. S11*, C and D*), hence indicating that the heterogeneous cross-sectional distribution of protofibrils could not explain alone the transition between the slow and fast regimes of lysis.

## Conclusion

Although the interplay between the main components of the fibrinolytic system is well understood, some dynamical aspects of the fibrinolysis remain unclear. For all experimental conditions, we observe that in vitro fibrin-rich clots undergo a slow and inefficient phase of degradation, characterized by a well-defined linear increase of turbidity, when subjected to endogenous fibrinolysis. This slow regime is always followed by a fast lysis regime, marked by a fast increase of turbidity. Although the durations of the slow and fast lysis regimes are in general correlated, their relative contributions depend on the experimental conditions and display opposite trends depending on the varying experimental parameter. Overall, we found that a large part of the lysis, if not most of it, operates in this slow regime regardless of the experimental conditions. To explain this observation, we proposed a computational model in which the properties of the binding of the proteins change during the lysis. First, Pg and tPA bind at the surface of the fibers, resulting in a slow lysis, then in the bulk of the fibers, hence speeding up the degradation of the clot. The model of fibrinolysis is complemented by a model of clot formation that aims to estimate the initial conditions for the concentrations of bound proteins and the radius of the fibers for the fibrinolysis model. Overall, the model of clot formation reasonably matches the experimentally measured radius of the fibers but seems to be less accurate than the model of fibrinolysis. This was somehow anticipated, first because of the simplifications we made in this model and second because of the lack of in-depth knowledge of the kinetic parameters. Having a model for clot formation able to reproduce the microstructure of a centimetric clot (or more) over a period of about 20 min would be of great interest but is a hard challenge that remains out of the scope of this work.

Our model of fibrinolysis differs from the seminal work of Diamond and Anand (25), as well as the latest works by Piebalgs, Xu, and co-workers (45,46), because we consider here that the number of binding sites accessible for the adsorption of soluble species dynamically changes during the lysis. We hypothesize that this mechanism allows us to recapitulate the different regimes of lysis as observed in our experiments. Identifying such transitions from surface to bulk reactions may be difficult to observe experimentally. However, after lysis of a fiber in real time, Feller et al. (54) discussed a phenomenological fiber-level model of fibrinolysis, with different timescales based on the integrity of the fibers. In their work, they proposed that the *α*C domains are cleaved by plasmin during the first stage of the lysis. Because these domains are responsible for interprotofibril interactions, the fibers start loosening, expose more binding sites, and facilitate the diffusion of plasmin through the fibers and, eventually, the binding of plasmin on the protofibrils. Then, a rapid phase of lysis follows, due to axial fragmentation of the protofibrils. In their work, the authors directly used plasmin, whereas in our case, we used tPA and Pg. However, the diffusion constants of plasmin, Pg, and tPA are quite similar (16); thus, if plasmin can diffuse more easily (according to Feller et al.), it might also be the case for tPA and Pg. Our hypothesis of dynamical change of binding sites accessibility may implement a coarse-grained version of the aforementioned phenomenological mechanism. Along the same lines, Li et al. (55) observed that plasmin-mediated lysis resulted in loosening and opening up the internal structure of a fiber, whereas stretching of a single fiber hampered its lysis by limiting access to cleavage sites. Our results indicated that heterogeneous cross-sectional distribution of protofibrils did not seem to be sufficient to explain alone the transition between the two regimes in a fibrin-rich clot. However, it would be worth investigating in future works whether the fractal lateral structure of the fiber plays a role in the intrinsic nature of the transition.

Previous works such as ours suffer from the assumption of a homogeneous shrinkage of the fibers, whereas the lysis of fiber instead occurs with transverse cutting across the surface of the fiber. Unfortunately, such an assumption has to be made to describe macroscopic lysis of centimetric clots with a duration of hundreds of minutes. Our description in term of number of shells involved in the kinetics of the lysis is strongly associated with the idea of uniform degradation. However, considering that lysis is facilitated because of easier access to binding and cleavage sites is not in contradiction with the formulation in terms of transverse cuts. A better understanding of dynamical change during lysis of protofibrils packing would certainly be of great interest for further experimental and theoretical works.

## Author contributions

F.R., K.Z.B., and B.C. designed the study and wrote the manuscript. F.R. developed the numerical models and performed data analysis. K.Z.B. and A.R. performed experiments. D.M. and D.P.-M. provided scanning electron microscopy images and performed scanning electron microscopy image analysis. K.Z.B. and B.C. supervised the project.

## Supporting citations

Reference (56) appears in the Supporting material.

## Supporting material

Document S1. Supporting materials and methods, Figs. S1–S11, and Table S1

Document S2. Article plus supporting material
