# Supplementary material for: Investigating the two regimes of fibrin clot lysis: an experimental and computational approach
Source: Biophys J. 2021 Aug 10;120(18):4091–106. doi: 10.1016/j.bpj.2021.08.005 (PMC8510862; doi:10.1016/j.bpj.2021.08.005)
Supplement: Document S1. Supporting materials and methods, Figs. S1–S11, and Table S1 [file mmc1.pdf]

Biophysical Journal, Volume *120*

Supplemental information

Investigating the two regimes of fibrin clot lysis: an experimental and computational approach

Franck Raynaud, Alexandre Rousseau, Daniel Monteyne, David Perez-Morga, Karim Zouaoui Boudjeltia, and Bastien Chopard

Investigating the two regimes of fibrin clot lysis: an experimental and computational approach

Franck Raynaud[1,*], Alexandre Rousseau[2], Daniel Monteyne[3,4], David Perez-Morga[3,4], Karim Zouaoui Boudjeltia[2], and Bastien Chopard[1]

[1]Department of Computer Science, University of Geneva, 1204 Geneva, Switzerland
[2]Laboratoire de Médecine Exépribmentale, Medicine Faculty, Université libre de Bruxelles (ULB 222 Unit), ISPPC CHU de Charleroi, Hôpital A. Vésale. Montigny-le-Tilleul, Belgium
[3]Laboratory of Molecular Parasitology, IBMM, Université libre de Bruxelles, Gosselies, Belgium
[4]Center for Microscopy and Molecular Imaging (CMMI), Université libre de Bruxelles, Gosselies, Belgium
[*]Correspondence: franck.raynaud@unige.ch

Supplementary Material

Experimental protocols

Proteins for experiments (Fibrinogen, tPA, PAI-1 and Plasminogen) were diluted in Owren-Koller buffer (DIAGNOSTICA STAGO®, France). Stock solutions were at -80° before utilization (Fibrinogen: 50mg/ml); tPA: 20μg/ml ; PAI-1: 25μ/ml ; Plasminogen : 1300μg/ml).
The clot formation started when 100 μl of thrombin (1.5 U/ml, DIAGNOSTICA STAGO®, France) were added.
For the semi-automatic clot formation and lysis measurement, the sample (mix of proteins) is introduced in a microcuvette and put in the apparatus, in a well. Then, the operator follows three operations to start the tests:

1 identification of sample

2 add thrombin

3 push on set button to start the recording of fibrinolysis process (5 sec max between the thrombin addition and the start of recording).

The device composition is:

- a thermostatic bloc (37°C) with eight photometric measure channels.
- a programmable language control (PLC)
- a computer and a software for the mathematical analysis of the fibrinolysis process

The thermostatic bloc is made of pure aluminium (360 × 30 × 100 mm) and is warmed by two resistances of 20 W. It is designed to insert spectrophotometric micro-cuvets (10 × 4 × 45 mm Sarstedt®) into 8 wells. Each well includes one emitter (diode: SFH 409) and one receptor (phototransistor: SFH309FA), both operating at 890 nm.

Programmable Language Control: ED&A V10, Antwerpen, Belgium. The sensors are connected to the PLC which transforms the analogic data (0–10 V) into digital data (12 bits). The data are transmitted to the computer by RS232.
The computer records every one minute the data from each channel. The software (developed with Windev® 4.1, France) generates the graph of the fibrinolytic process. The design of a lysis curve is exemplified in Figure 1A. The x-axis/y-axis respectively represent time and evolution of the signal sensor. Mathematical analysis or complete procedure can be started individually for each sample and at every moment without disturbing the measurements of other samples. The final assembly was performed by EREM, (Marcinelle, Belgium) and the system is distributed since 2001 by BIOK, (Montigny-Le-Tilleul, Belgium).

Electronic microscopy procedure

For the electronic microscopy, the fibrin clots were formed from Fibrinogen ($400\mu l$) mixed with thrombin $100\mu l$ (1,5 U/ml). Samples were placed at 37°C, 10 min. Then clots were fixed with buffer glutaraldehyde (2,5%) cacodylate buffer (0.01M) during 60 min. Clots were washed three times with only cacodylate buffer before scanning electronic microscopy procedure. External faces of clots were analyzed in first intention. Clots were also cut to observe the fibrin structure inside.

Model of clot formation: range of values of the kinetic parameters

Parameter	Definition	Range of values	Unit
k_{PI}	protofibril initiation	$10^{-19}, 10^{-18}, \mathbf{10^{-17}}, 10^{-16}, 10^{-15}$	$L \cdot (molecule \cdot s)^{-1}$
k_{FG}	fiber growth	$\mathbf{10^{-16}}, 10^{-15}, 10^{-14}$	$L \cdot (molecule \cdot s)^{-1}$
k_{FI}	fiber initiation	$10^{-22}, 10^{-21}, 10^{-20}, 10^{-19}, \mathbf{10^{-18}}$	$L \cdot (molecule \cdot s)^{-1}$
k_A	fibrinopeptide A cleavage	$0.001, 0.002, 0.004, 0.008, 0.01, \mathbf{0.02}, 0.04$ $0.08, 0.1, 0.2, 0.4, 0.8$	s^{-1}
k_{FA}	fiber aggregation	$10^{-20}, 10^{-19}, \mathbf{10^{-18}}, 10^{-17}, 10^{-16}, 10^{-15}, 10^{-14}$	$L \cdot (molecule \cdot s)^{-1}$
k_{PG}	protofibril growth	$10^{-18}, 10^{-17}, 10^{-16}, \mathbf{10^{-15}}, 10^{-14}$	$L \cdot (molecule \cdot s)^{-1}$
Fibrinogen		$1, 2, 3, 4$	$mg \cdot ml^{-1}$

Table S 1: Range of values of the kinetic parameters used in the model of clot formation. All possible sets of parameters were tested using these values, thus resulting in more than 31000 sets of parameters. Among all these possible sets, 92 could reproduce the values of R_f observed experimentally. These 92 sets were tested, compared with experimental lysis curves and ranked based on the maximal error between simulated and experimental lysis profiles. All simulations presented in this work were made with the set of parameters that received the best score. These values are indicated in bold. For the variance-based sensitivity analysis, the min and the max of each parameter were used to generate samples, with a concentration of fibrinogen of $3mg.ml^{-1}$.

Supplementary Figures

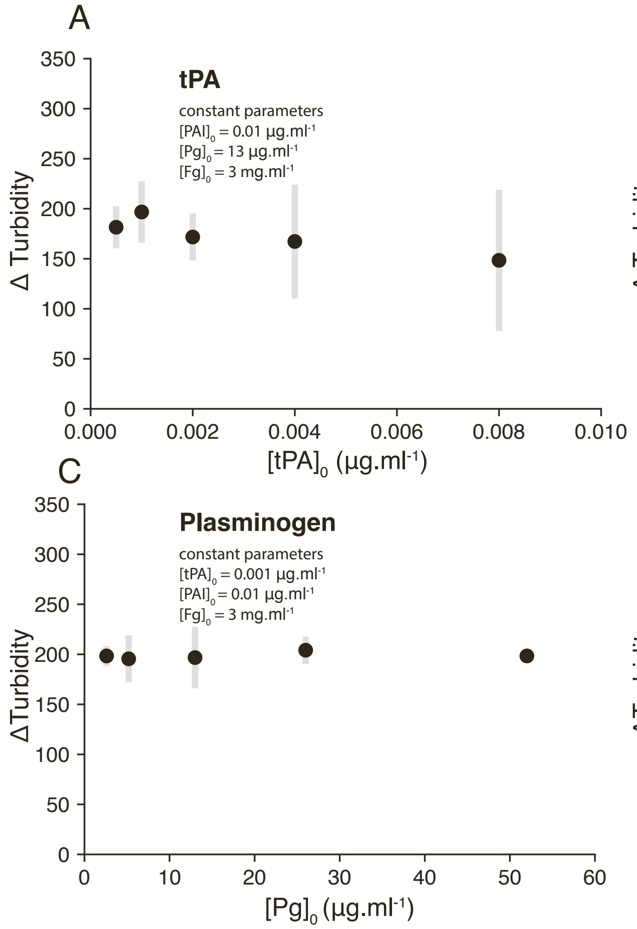

Figure S 1: Turbidity and concentration of fibrinogen are correlated. Mean drop of turbidity (Δ Turbidity) for different concentrations of A) tPA, B) PAI-1, C) Plasminogen and D) Fibrinogen. The bars represent the standard deviation. Only one experimental condition was varied at a time while all the others remained constant (the constant parameters are listed on each panel).

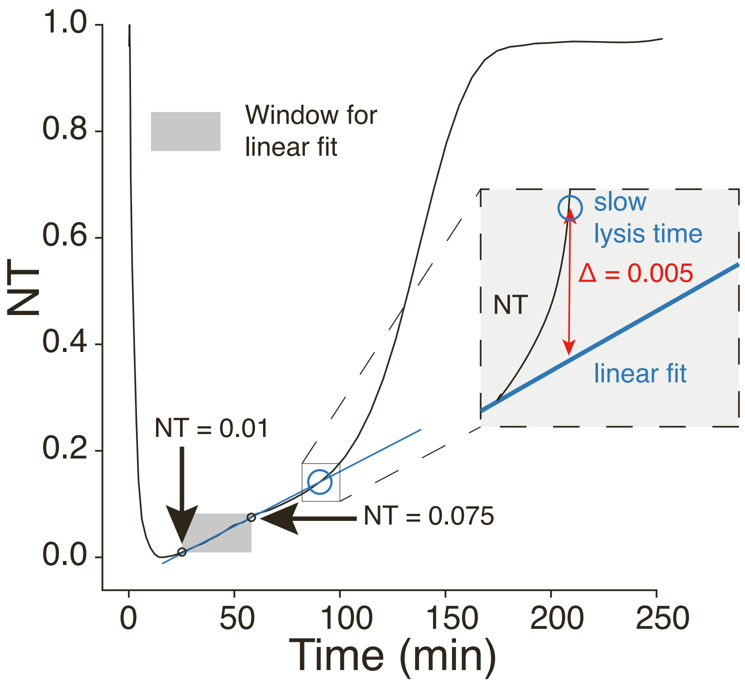

Figure S 2: Determination of the slow lysis time. First we find the two points with NT values 0.01 and 0.075 as well as their corresponding time. These two points define the window where the linear fit is made (gray window). After fitting, the NT is browsed from its end until the NT curve is distant from the fit by a value $\Delta = 0.005$ (red double arrows in the inset). This point marks the slow lysis time (blue empty circle).

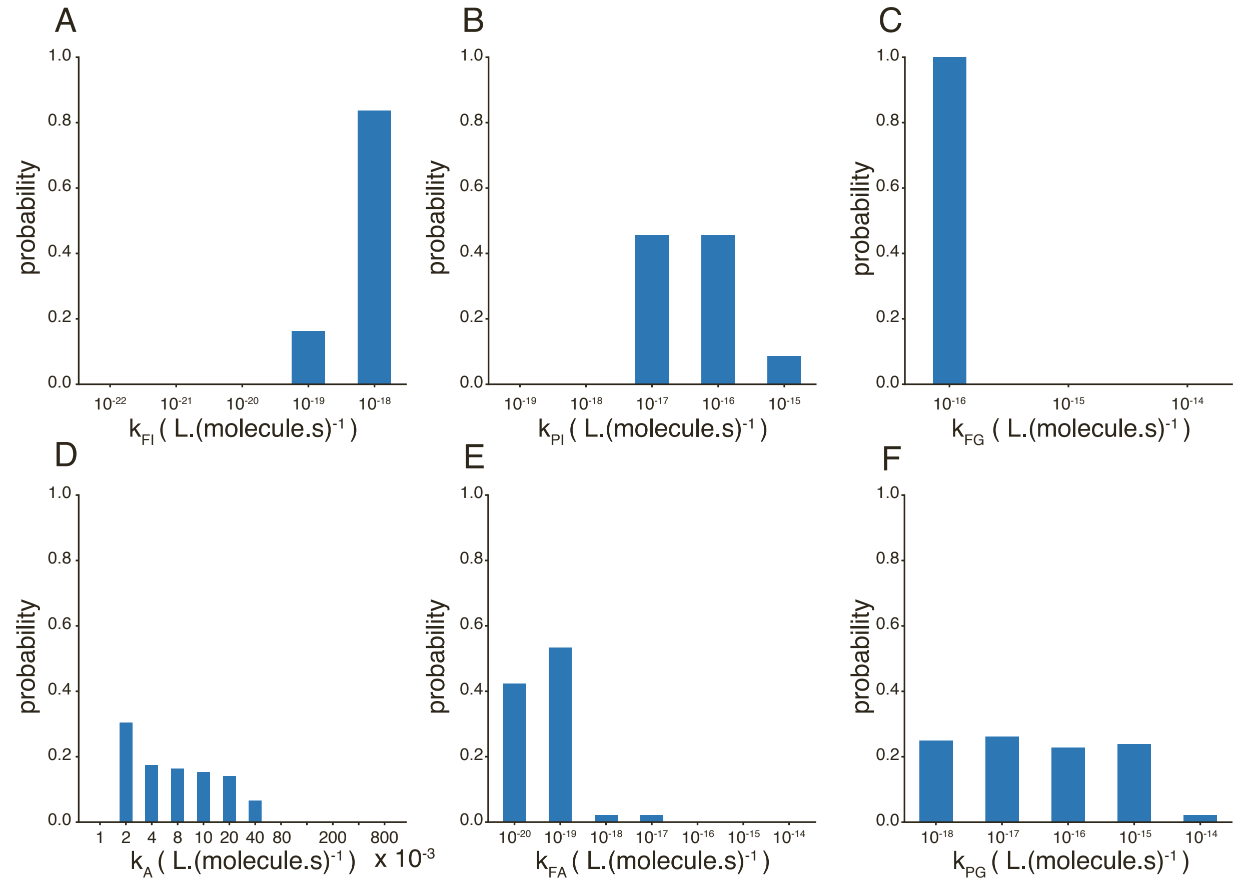

Figure S 3: Marginal probability of the kinetic parameters of the clot formation model in the 92 sets of parameters. A) Fiber initiation k_{FI}. B) Fibrin monomer association k_{PI}. C) Addition of protofibrils to growing fiber. D) Cleavage of fibrinopeptide A. E) Fiber-fiber aggregation k_{FA}. F) Growth in length of protofibrils k_{PG}.

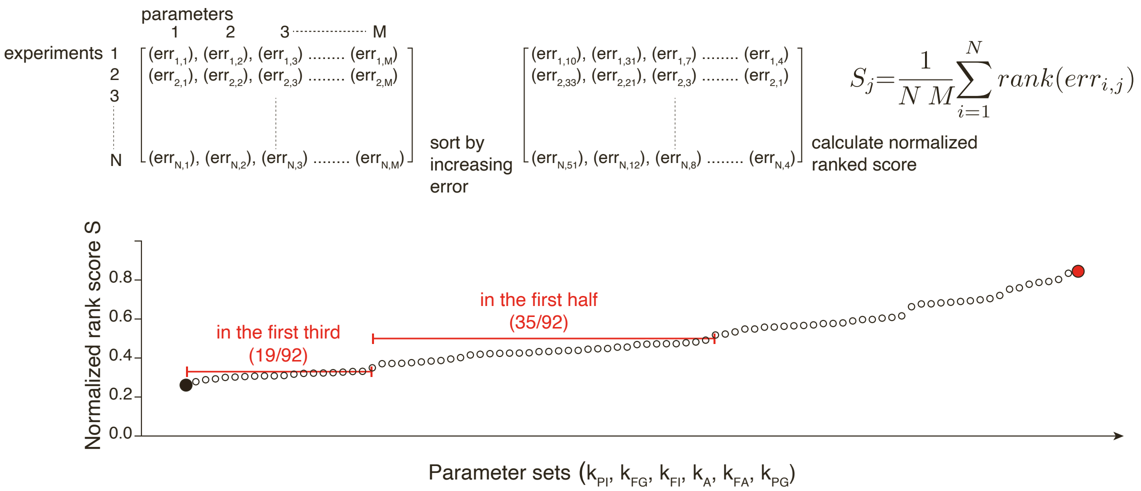

Figure S 4: Scoring of the kinetic parameters of the clot formation model and methodology to determine the score of each set of kinetic parameters. First we measured err_{ij} the maximum error between simulated and experimental lysis profile for an experiment i and a set of parameters j. Then we sorted the errors for each experimental condition and summed their corresponding rank to calculate the score S (upper panel). Distribution of the score for all the parameters sets (lower panel) with the best (black dot) and worst (red dot) ranked parameters sets.

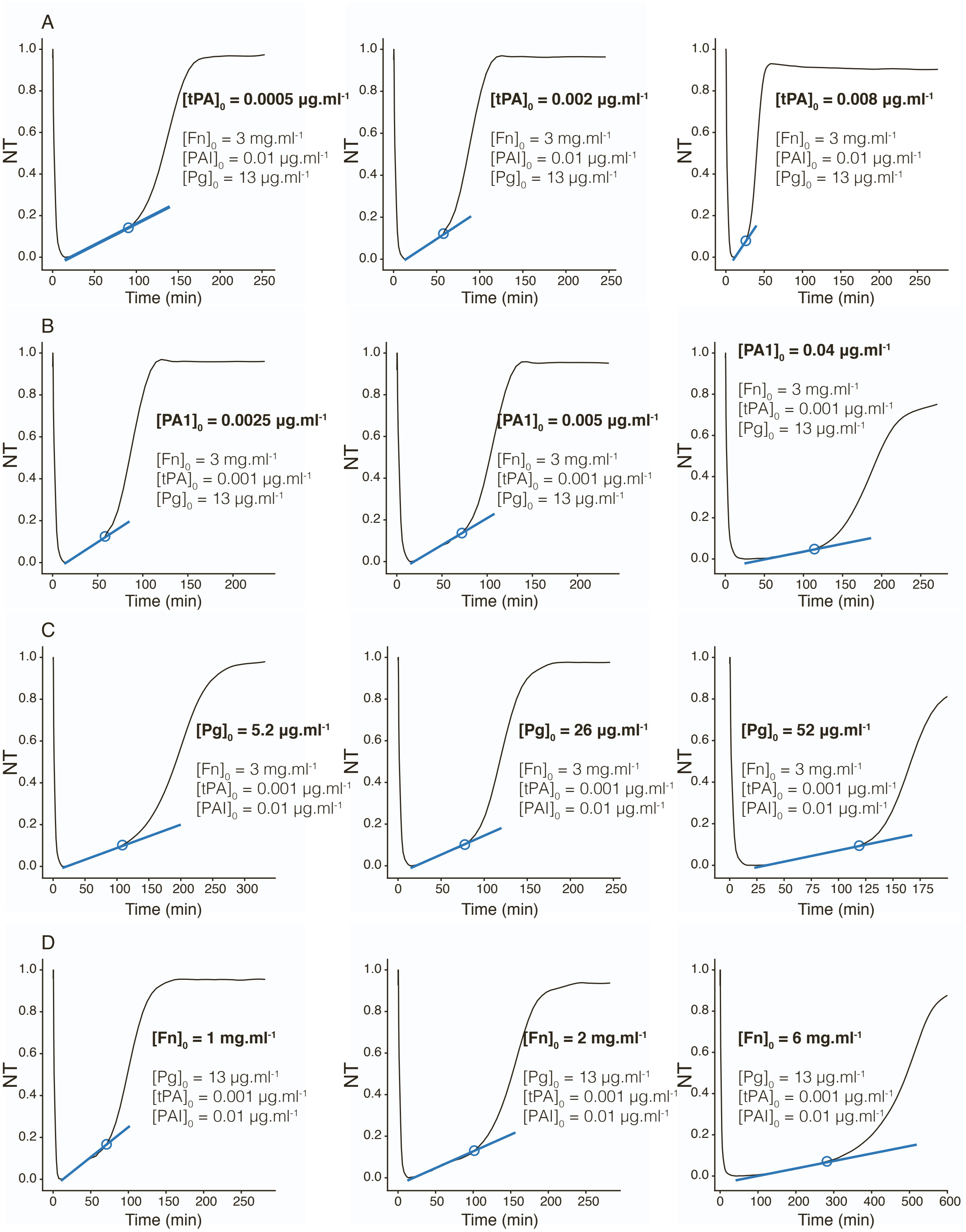

Figure S 5: Linear increase in time of the normalized turbidity (NT) during the slow regime, and normalized turbidity time curve for different concentrations of A) tPA, B) PAI-1, C) Plasminogen, D) Fibrinogen.

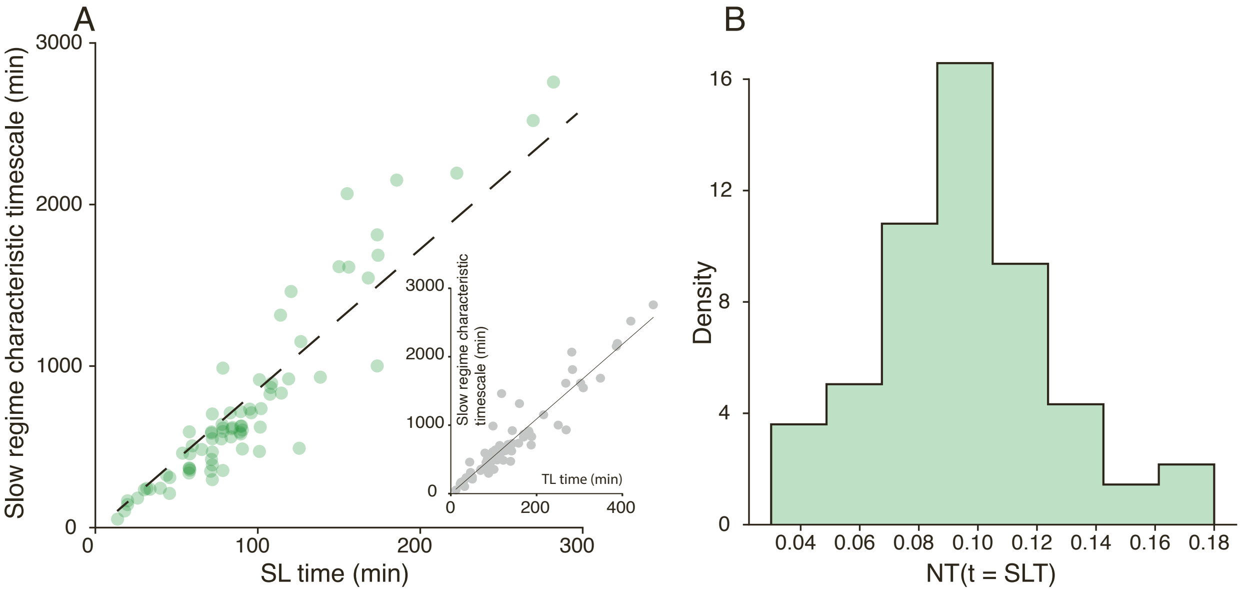

Figure S 6: Correlation between the inverse of the slope of the linear fit and the SL time. A) Inverse of the slope of the linear fit as a function of SL time (inset: TL time). B) Normalized histogram (density) of the values of the normalized turbidity (NT) at the end of the slow regime.

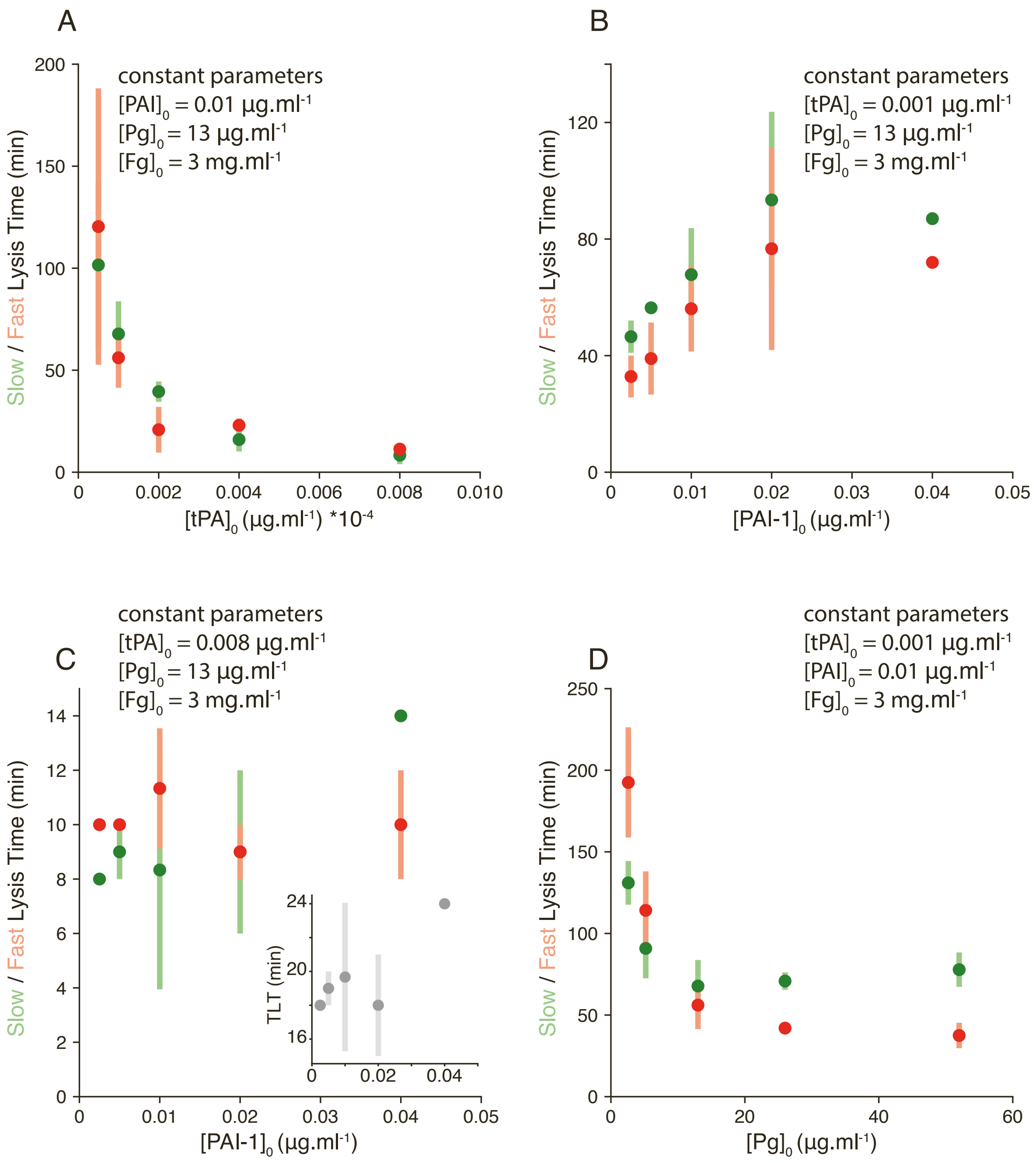

Figure S 7: Mean slow and fast lysis times for different concentrations of A) tPA, B) PAI-1 and $[tPA]_0 = 0.001\mu.ml^{-1}$, C) PAI-1 and $[tPA]_0 = 0.008\mu.ml^{-1}$ (inset: total lysis time, TLT), D) Plasminogen. Bars represent the standard deviation. Only one experimental condition was varied at a time while all the others remained constant.

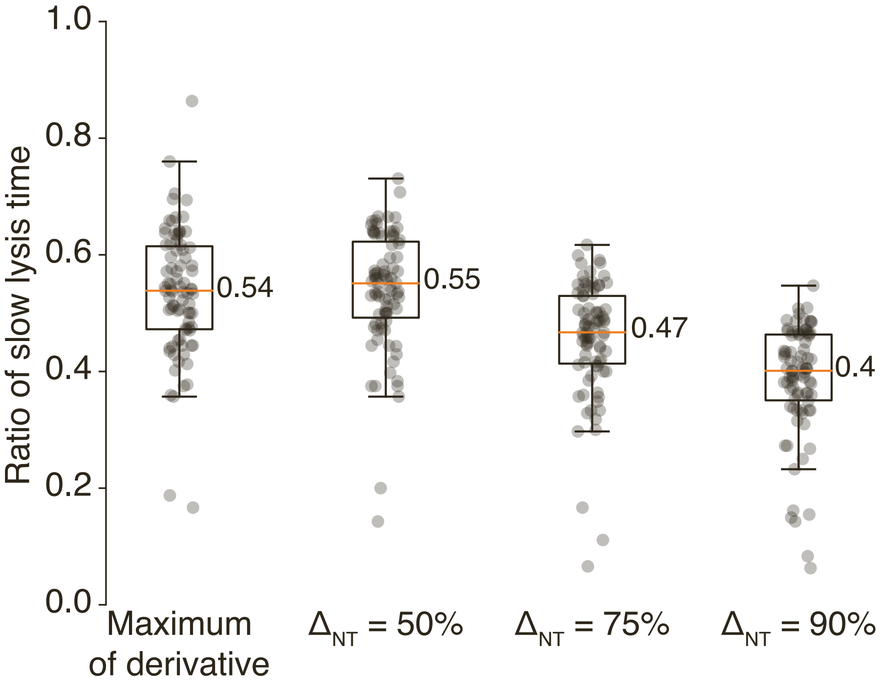

Figure S 8: Ratio of the slow to the total lysis time for different definitions of the lysis time: the time point where maximum of the derivative of the turbidity curve, 50% of turbidity change, 75% of turbidity change and 90% of turbidity change. The boxplots represent the median as well as 1st and 3rd quartiles, median is indicated on the figure.

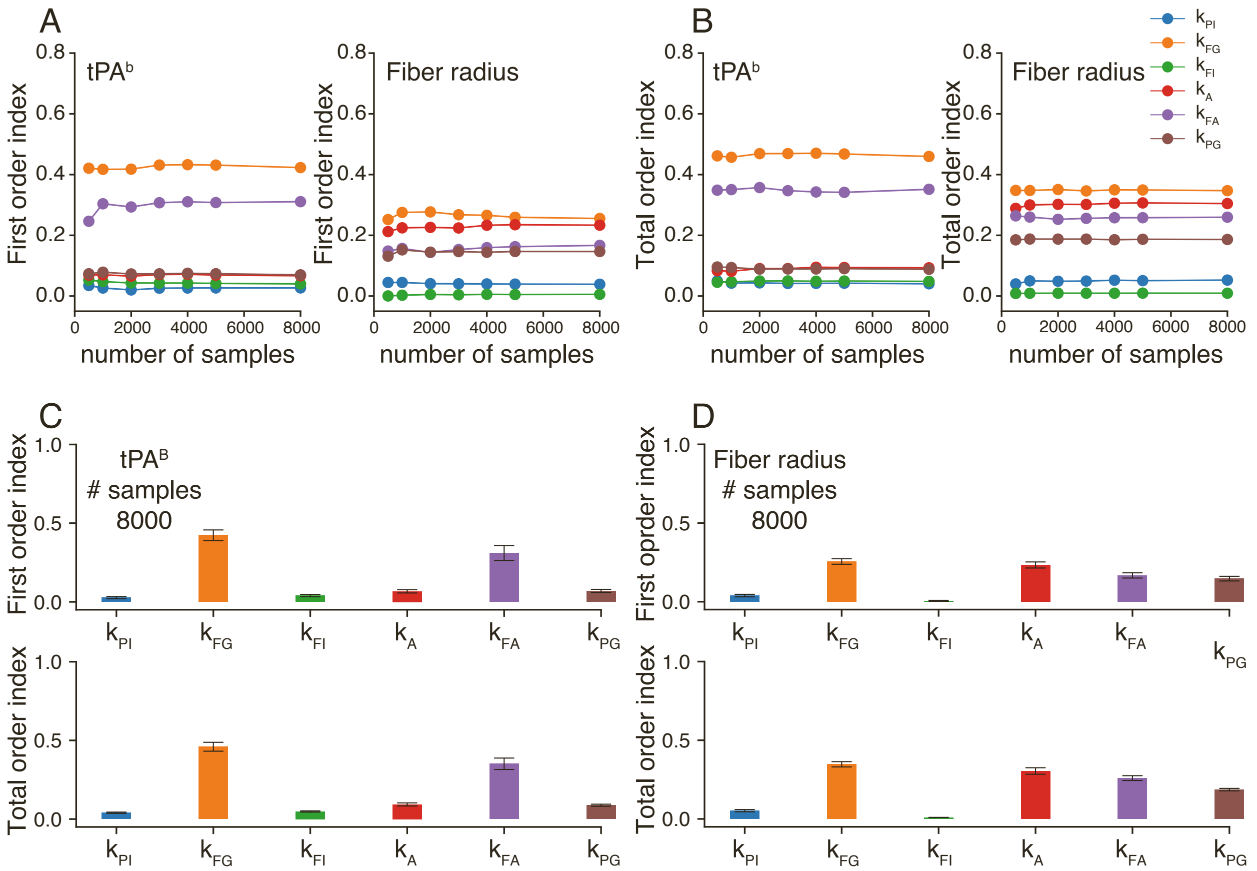

Figure S 9: Variance-based sensitivity analysis for the clot formation model. A) Effect of the sample size on the values of the Sobol first order indices for the concentration of tPA^b (left panel) and R_{f0} (right panel). B) Effect of the sample size on the values of the Sobol total order indices for the concentration of tPA^b (left panel) and R_f (right panel). The values of both first and total order indices converge as the number of samples increases. C) Sobol first order indices (upper panel) and total order indices (lower panel) for 8000 samples for the concentration of tPA^b. D) Sobol first order indices (upper panel) and total order indices (lower panel) for 8000 samples for R_f. The higher is the first (resp. total) order index, the more important is the contribution of the parameter for the total variance by its single (resp. by its interactions with others) effect. The variance-based sentivity analysis was done using the SALib python library [1]. Samples were generated with Saltelli's sampling scheme (uniform distributions of the parameters) using the parameters given in Table S 1 and a concentration of fibrinogen $[Fg] = 3mg.ml^{-1}$.

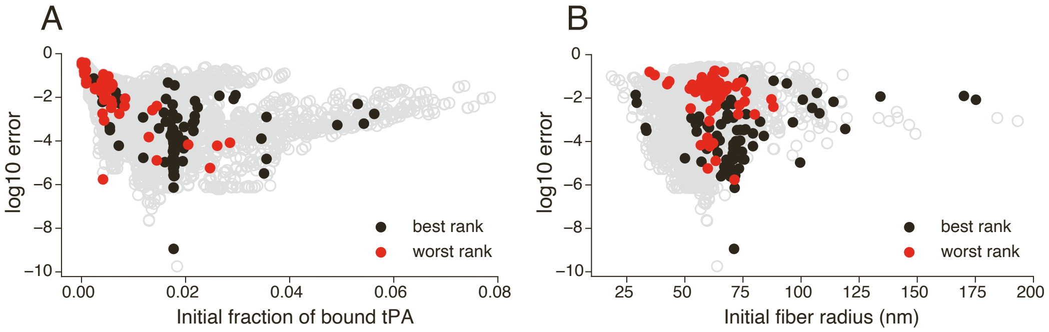

Figure S 10: A) Error between the simulations and the experiments as function of the initial concentration of tPA^b for all the experimental conditions with the best (black dots) and worst (red dots) ranked parameters sets. Concentration of tPA^b are similar within the best and worst sets of parameters, but different among them, indicating that for the best set of parameters around 2% of the initial concentration of tPA bound to the fibers. B) Same as A) for the initial radius of the fibers R_{f0}. Black (resp. red) dot represents a simulation of an experiment with the best (resp. worst) ranked set of parameters, the y-axis is the log10 of the mean squared error. Gray dots represent other parameters sets.

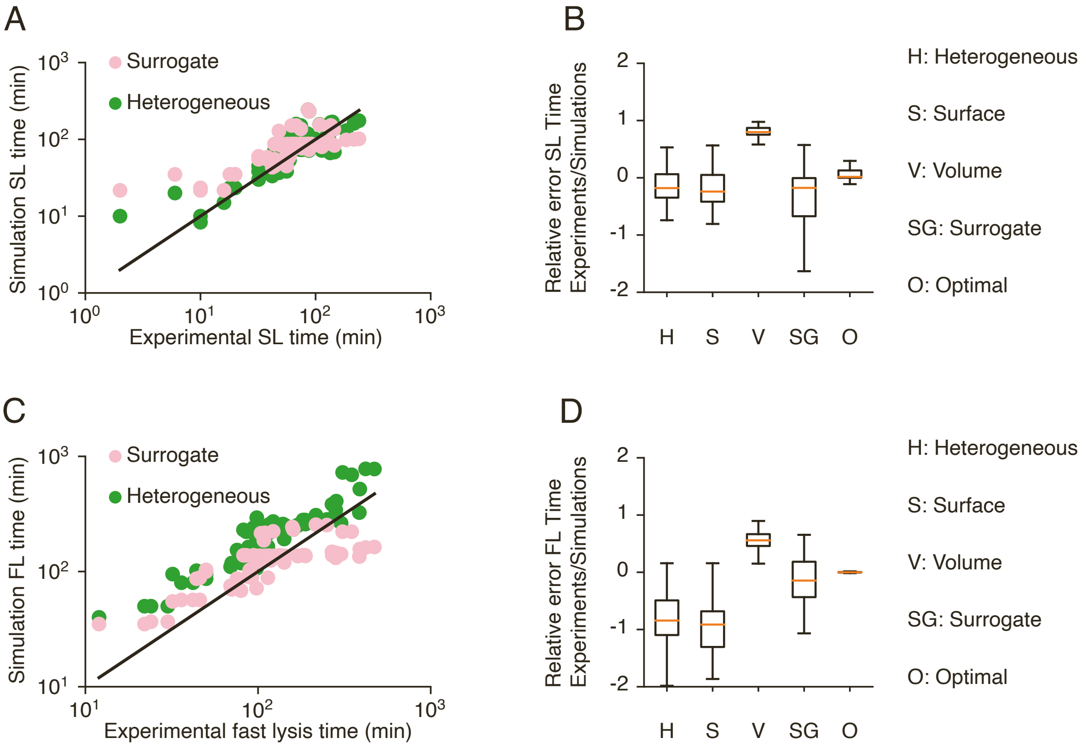

Figure S 11: Slow and fast lysis times for a model with heterogeneous cross-sectional distribution of protofibrils. A) Comparison of experimental and simulated slow lysis times for the surrogate model (pink) and the heterogeneous model (green). B) Comparison of the relative errors between the experimental and simulated slow lysis times for the different models. C) Comparison of experimental and simulated fast lysis times for the surrogate model (pink) and the heterogeneous model (green). D) Comparison of the relative errors between the experimental and simulated fast lysis times for the different models. The boxplots represent the median as well as 1st and 3rd quartiles. Simulations for the heterogeneous model were made with the same parameters as other models. The number of outer protofibrils was calculated from the results given in [2].

Supporting References

[1] Jon Herman and Will Usher. SALib: An open-source python library for sensitivity analysis. *The Journal of Open Source Software*, 2(9), jan 2017.

[2] Wei Li, Justin Sigley, Stephen R. Baker, Christine C. Helms, Mary T. Kinney, Marlien Pieters, Peter H. Brubaker, Roger Cubcciotti, and Martin Guthold. Nonuniform Internal Structure of Fibrin Fibers: Protein Density and Bond Density Strongly Decrease with Increasing Diameter. *BioMed Research International*, 2017, 2017.
